# Metal-mediated aminocatalysis provides mild conditions: Enantioselective Michael addition mediated by primary amino catalysts and alkali-metal ions

**DOI:** 10.3762/bjoc.9.18

**Published:** 2013-01-23

**Authors:** Matthias Leven, Jörg M Neudörfl, Bernd Goldfuss

**Affiliations:** 1 Department of Chemistry, Universität zu Köln, Greinstrasse 4, D-50939 Köln, Germany, Fax: +49(0)221-470-5057

**Keywords:** alkali metals, aminocatalysis, DFT-calculations, Lewis acids, Michael addition

## Abstract

Four catalysts based on new amides of chiral 1,2-diamines and 2-sulfobenzoic acid have been developed. The alkali-metal salts of these betaine-like amides are able to form imines with enones, which are activated by Lewis acid interaction for nucleophilic attack by 4-hydroxycoumarin. The addition of 4-hydroxycoumarin to enones gives ee’s up to 83% and almost quantitative yields in many cases. This novel type of catalysis provides an effective alternative to conventional primary amino catalysis were strong acid additives are essential components.

## Introduction

Organocatalysis based on primary or secondary amines enables a myriad of enantioselective transformations, e.g., with aromatic or aliphatic enones [1]. There are many approaches employing protonated derivatives of chinchona alkaloids [2–5], amino acid derivatives, and chiral 1,2-diamines, which provide excellent yields and enantioselectivities. Among the most famous examples are Mac Millan’s secondary-amine-based catalysts [6] or 9-amino-9-deoxy-epihydroquinine salts used by Jørgensen et al. [7–8].

Another growing field of catalysis is metal-promoted organocatalysis, which is closely related to tandem reactions of metal- and organocatalysis [9–13]. In most cases, transition or rare-earth metals are employed as Lewis acids in order to activate electrophilic compounds to react with substrates that are directly bonded to an organic catalyst [9–13]. An example is the enamine–metal Lewis acid bifunctional catalysis for asymmetric direct aldol reaction, which is mediated by copper ions and amino acid derivatives [14] or enamine nucleophilic addition to palladium π-allyl electrophiles [15–17].

**Scheme 1:**
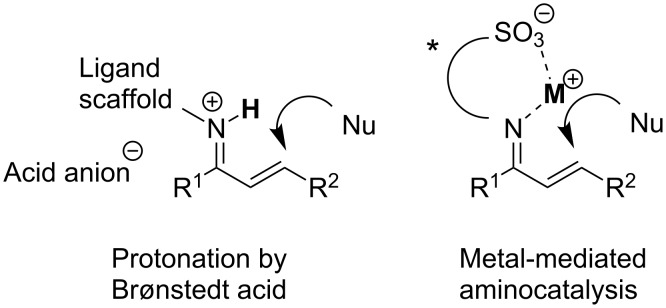
Activation of amine-bonded Michael acceptors by protonation versus Lewis acid interaction.

We report the development of new catalysts based on chiral 1,2-diamines and present their application in the asymmetric addition of 4-hydroxycoumarin (**1**) to prochiral α,β-unsaturated ketones. Instead of acid additives, which sometimes lead to decomposition of sensitive components [18], alkali-metal ions are employed as very mild Lewis acids (Scheme 1).

**Scheme 2:**
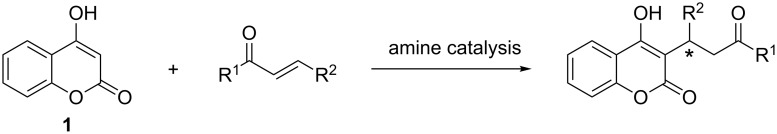
Synthesis of 4-hydroxycoumarin derivatives by Michael addition [19–27].

The activation of Michael acceptors by iminium ions enables asymmetric additions of 4-hydroxycoumarins to α,β-unsaturated ketones (Scheme 2) [19–27].

There is a wide spread of 4-hydroxycoumarins in pharmaceuticals such as anticoagulants and substances that inhibit HIV or malaria [28–29]. Among the most prominent chiral 4-hydroxycoumarins is warfarin, which works as a vitamin K antagonist, and the dissimilar activity of the enantiomers is well documented in the literature [30–32]. The organocatalytic synthesis of warfarin has already been tested with several organocatalysts. Diphenylglycinol derivatives and derivatives of 1,2-diphenylethane-1,2-diamine provided good yields and ee’s in the range of 80% [19–27]. Other attempts employing chinchona alkaloids provided ee’s higher than 90% [24].

## Results and Discussion

**Scheme 3:**
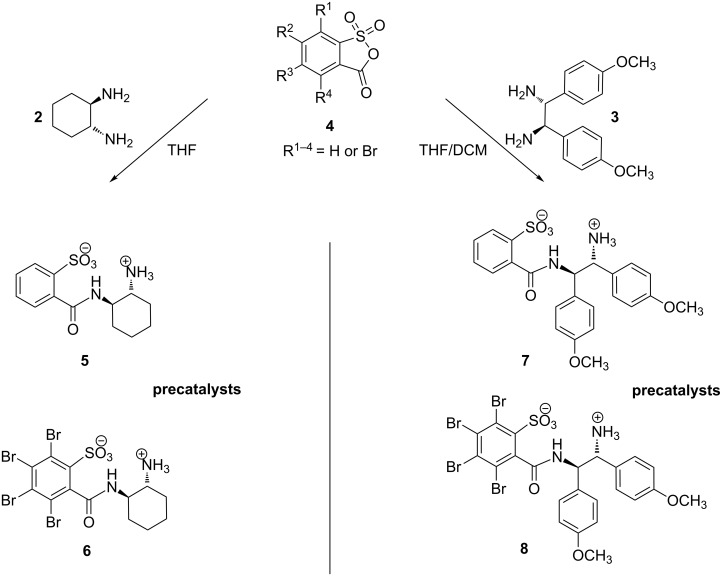
Precatalysts **5**–**8** and synthesis from chiral 1,2-diamines and 2-sulfobenzoic anhydrides.

The newly developed catalysts are derived from *C*_2_-symmetric chiral diamines **2** and **3** and 2-sulfobenzoic acid by formation of a carboxylic amide (Scheme 3). The active site consists of a primary amino group and a sulfonic acid group, which can form chelates with cations. The sulfonic acid derivatives are accessible through efficient transformations of diamines with 2-sulfobenzoic anhydrides **4** (Scheme 3).

**Figure 1:**
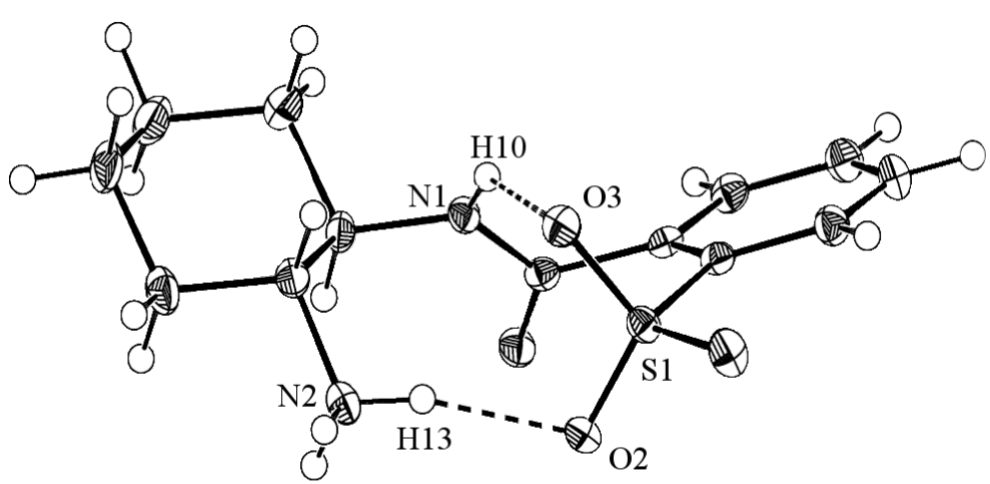
X-ray crystallographic structure of **5**. The conformation of the 2-sulfobenzoic moiety is fixed by hydrogen bonds. The distance H10**^…^**O3 is 2.283 Å, the distance H13**^…^**O2 is 1.920 Å. (distance N1-O3: 2.865 Å, distance N2-O2: 2.822 Å, ellipsoids: 50% probability)

Typical yields of the transformation (Scheme 3) are 83% for **5** and 75% for **7**. Side-products such as the diamides are rarely formed if an immediate in situ precipitation of the desired product is achieved. The structural chelate character of the spontaneously formed salts can also be observed in the crystallographic structure of **5** (Figure 1). A characteristic detail in the X-ray crystallographic structure of **5** is the intramolecular hydrogen bond between H10 and O3 (distance: 2.283 Å) in Figure 1, which restricts the conformation of the 2-sulfobenzoic moiety.

It was found that the solubility of each precatalyst shown in Scheme 3, which is essential for the application in catalysis, depends strongly on the specific substituents. The 1,2-diaminocyclohexane-based precatalyst **5** is soluble in water but hardly in methanol or THF if no DMSO is added. Thus, the application of this compound in catalysis is limited by the range of appropriate solvents. Bromine derivative **6**, which is based on 1,2-diaminocyclohexane as well, exhibits a markedly higher solubility in THF and can also be dissolved in mixtures of alcohols and DCM. Precatalysts **7** and **8** are derived from 1,2-diphenylethane-1,2-diamine and the solubility of these substances in organic solvents is significantly higher than that of **5** or **6**. This effect could be due to the methoxy groups, and **8** can easily be applied in THF or chloroform.

**Figure 2:**
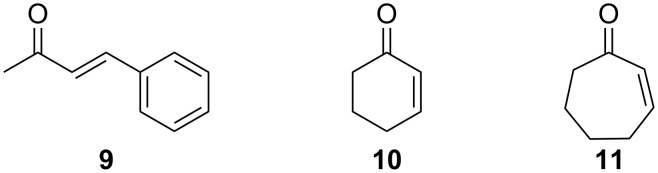
Michael acceptors employed as substrates in the nucleophilic addition of 4-hydroxycoumarin (**1**).

The first step in finding a well-performing catalytic active system was to find a catalyst–metal combination that generates satisfying yields. The idea was to choose a Lewis acid that is strong enough to activate the imine preformed by the catalyst and the Michael system, so that the nucleophilic attack shown in Scheme 1 occurs. On the other hand Lewis acids that are too strong could inhibit the formation of the imine by complexation of the primary amino group. The most promising counter ions are expected to be from the first group of the periodic table of elements, and test reactions were carried out by employing catalyst **5** and **7** with benzylideneacetone (**9**) and cyclohex-2-enone (**10**) (Figure 2) as substrates (Table 1). It was found that very mild conditions prevail under catalytic conditions if alkali metals are used as Lewis acids, since the pH value of the dissolved catalyst is in the range of 9 (see Experimental section for details).

**Table 1:** Screening of **5** and **7** with different metal counter ions.^a^

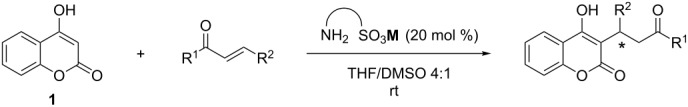					

Entry	Catalyst	M	Time [d]^b^	Yield [%]^c^	ee [%]^d^

1	**5**	H	3	<5	–
2	**7**	H	3	<5	–
3	**5**	Li	2	94	44
4	**7**	Li	2	94	15
5	**5**	Na	3	72	47
6	**7**	Na	3	7	26
7	**5**	Zn	4	0	–
8	**7**	Zn	4	5	21
9	**5**	K	2	62	36
10	**7**	K	2	74	28
11^e^	**5**	Na	3	45	47
12^e^	**7**	Na	3	99	16

^a^All reactions were carried out with 20 mol % catalyst in abs. THF/DMSO 4:1 at room temperature. Unless otherwise specified the substrate was benzylideneacetone (**9**). ^b^Conversion controlled by TLC. ^c^Isolated yield. ^d^ee determined by chiral HPLC. ^e^Substrate was cyclohex-2-enone (**10**).

The application of catalysts **5** and **7** (Table 1) indicates that lithium cations are the most suitable counterions for the activation of the Michael acceptor, with 94% isolated yield (Table 1, entries 3 and 4). Sodium and potassium ions are slightly less sufficient with yields up to 72% (sodium, Table 1, entry 5) or 74% (potassium, Table 1, entry 10). Zinc does almost not work at all (yields are 5% or less, Table 1, entries 7 and 8). The reason for the low yields when using zinc may be that the Lewis acidity is too strong, which could lead to formation of inactive complexes of the catalyst. Since hydrogen (M = H) does not provide any isolatable conversions at all (Table 1, entries 1 and 2), it seems to be clear that the zwitterionic chelate shown in Figure 1 is too inert to form imines with carbonylic compounds. The choice of alkali metal does not affect the ee very markedly and the ee’s obtained in Table 1 are in the moderate range (up to 47%).

In order to elucidate if Lewis acid free imines could also react as Michael acceptors, experiments employing triethylammonium salts of the catalysts **5** and **7** (pH 9) were performed, and no conversions could be detected in all cases.

Because lithium was identified as the most appropriate alkali-metal ion for application in the catalysis (Table 1), the following experiments were carried out with lithium salts of catalysts **5**–**8**. In order to improve the enantioselectivities, solvent related conditions were varied systematically so that reactions could be carried out at low temperatures. 4-Hydroxycoumarin and the catalysts require very polar solvents as additives, e.g., DMSO, which precipitate at low temperatures. Alcohols employed as antifreezing additives at temperatures of −20 °C or as pure solvents effected decreasing ee’s in the most cases. Thus, the range of suitable solvents was strongly limited by the low solubility of all compounds in indifferent liquids. Nevertheless, the ee could be improved up to 83% (Table 2, entry 7).

**Table 2:** Screening of catalysts **5**, **6**, **7**, **8** under various conditions with different substrates.^a,b^.

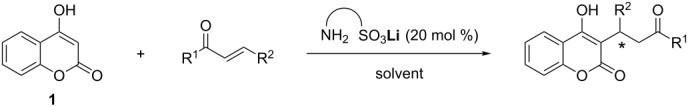							

Entry	Substrate^a^	Catalyst^b^	Solvent^c^	*T* [°C]	Time [d]	Yield [%]^d^	ee [%]^e^

1	**10**	**7**	1	0	3	99	72
2	**10**	**7**	2	−20	4	61	66
3	**10**	**7**	3	0	3	99	64
4	**10**	**7**	4	0	3	99	63
5	**10**	**5**	1	20	2	46	17
6	**10**	**7**	1	20	2	99	68
7	**10**	**8**	1	0	2	99	83
8	**9**	**6**	1	20	2	99	42
9	**11**	**8**	1	0	2	99	60
10	**11**	**7**	1	0	2	99	51
11	**10**	**6**	1	20	2	77	23
12	**10**	**8**	1	20	2	61	43
13	**11**	**5**	1	20	2	44	13
14	**11**	**6**	1	20	2	63	30

^a^**10** = Cyclohex-2-enone; **9** = benzylideneacetone; **11** = cyclohept-2-enone. ^b^The metal counter ion is lithium. ^c^Solvents: 1 = abs. THF/DMSO 4:1; 2 = abs. THF/ethylene glycol 4:1; 3 = dioxane; 4 = isopropanol. ^d^Isolated yield. ^e^ee determined by chiral HPLC.

The results shown in Table 2 indicate that the catalysts **7** and **8** based on 1,2-diphenylethane-1,2-diamine perform indeed better than **5** and **6**, based on *trans*-1,2-diaminocyclohexane: The highest ee that could be obtained by **8** is 83% whereas the *trans*-diaminocyclohexane derivatives could only reach up to 47% ee (Table 1, entry 5). There is a strong preference for **7** to produce higher ee’s if cyclic enones are used, whereas diaminocyclohexane derivatives perform better with the acyclic *trans*-enone **9** (Table 1, entry 4 versus Table 2, entry 1). There is also a significantly stronger chiral induction for the bromine derivative **8** derived from **7** (ee increases from 72% to 83%, Table 2, entries 1 and 7).

### The origin of enantioselectivity

**Scheme 4:**
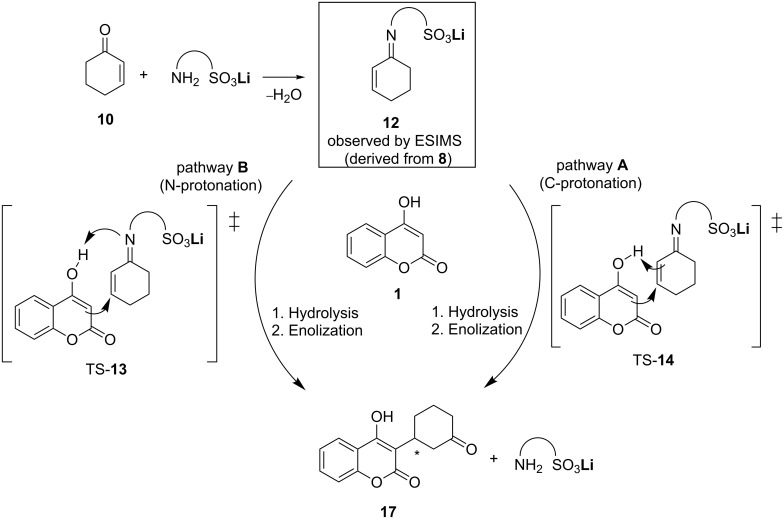
Computationally analyzed pathways **A** (C-protonation), and **B** (N-protonation), arising from the addition of 4-hydroxycoumarin to cyclohexenone.

In order to obtain a mechanistic insight into the newly designed catalysis and the origin of enantioselectivity, DFT calculations on the enantio-determining transition states were performed by employing the nonempirical TPSS-functional and BP86. Two possible pathways were found computationally (pathway **A**, **B**, Scheme 4).

The first essential step in the catalytic process is the formation of the imine intermediate **12** from the catalyst and the Michael substrate **10** (Scheme 4) [6]. Formation of **12** by catalyst **8** (Figure 2) under in situ conditions could also be proven by ESIMS spectrometry (*m*/*z* = 855.1, see Supporting Information File 1 for details) to underpin an activation process by the imine–lithium complex. The addition of 4-hydroxycoumarin to the activated Michael system **12** could now occur along two slightly different mechanistic pathways (**A**, **B**, Scheme 4) including the enantio-determining transition states corresponding to TS-**13** or TS-**14**. Both pathways require the formation of a carbon–carbon bond and the dissociation of the oxygen–hydrogen bond of the hydroxy group, exhibited by the 4-hydroxycoumarin. The only difference lies in the migration of the shifted proton to carbon (pathway **A**) or nitrogen (pathway **B**).

**Scheme 5:**
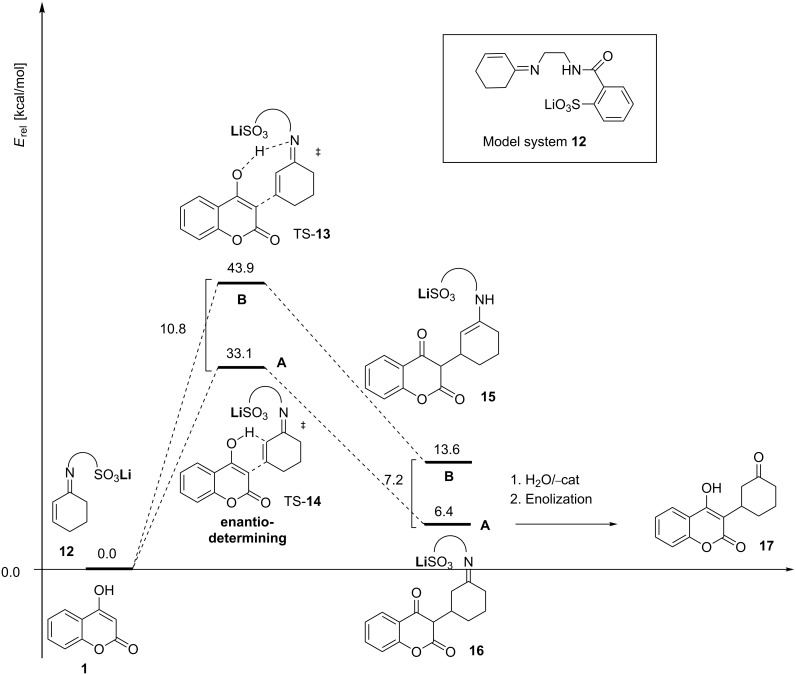
Computed energy profile for reaction path **A** (C-protonation) and **B** (N-protonation) corresponding to a model system **12** BP86/SVP+ZPE. The C-protonation route includes the more favored enantio-determining transition state TS-**14**. Hydrolysis and enolization of intermediates **15** and **16** will subsequently occur under in situ conditions.

Both reaction paths considered in Scheme 5 are of concerted character, and ionic intermediates of stepwise additions are not stationary points on the potential energy surface. The relative energies for the two possible pathways **A**, **B** (Scheme 5) suggest that the reaction path **A**, involving C-protonation in the enantio-determining step, is clearly favored. The enantio-determining transition states TS-**13** and TS-**14** differ by 10.8 kcal/mol, whereby TS-**14** is the favored. Intermediate **16** is 7.2 kcal lower in energy than **15**. Hence, it is apparent that the enantio-determining step of the addition to the Michael acceptor is TS-**14**. Another point of interest in the mechanistic study is how enantioselectivity can be predicted [33].

**Figure 3:**
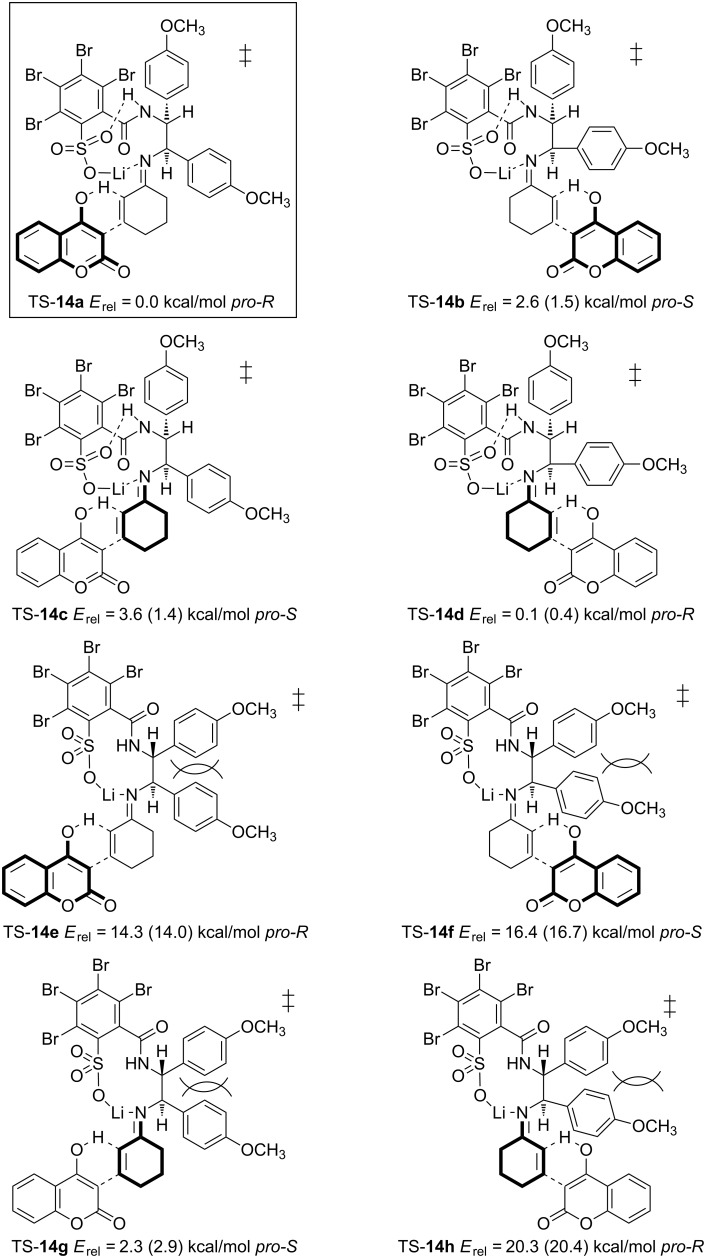
Enantio-determining transition states arising from the addition of 4-hydroxycoumarin to cyclohexenone employing **8**. Only transition states corresponding to TS-**14** (pathway **A**) are shown, TPSS/SVP+ZPE (BP86/SVP+ZPE).

In order to elucidate the mechanistic process, all accessible enantio-determining transition structures corresponding to TS-**14** were computed for the addition of 4-hydroxycoumarin to cyclohexenone employing ligand **8**. There are two conformational degrees of freedom (Figure 3) giving rise to eight structures (four *pro-R* and four *pro-S*-configurations), which have to be compared (Figure 3).

**Figure 4:**
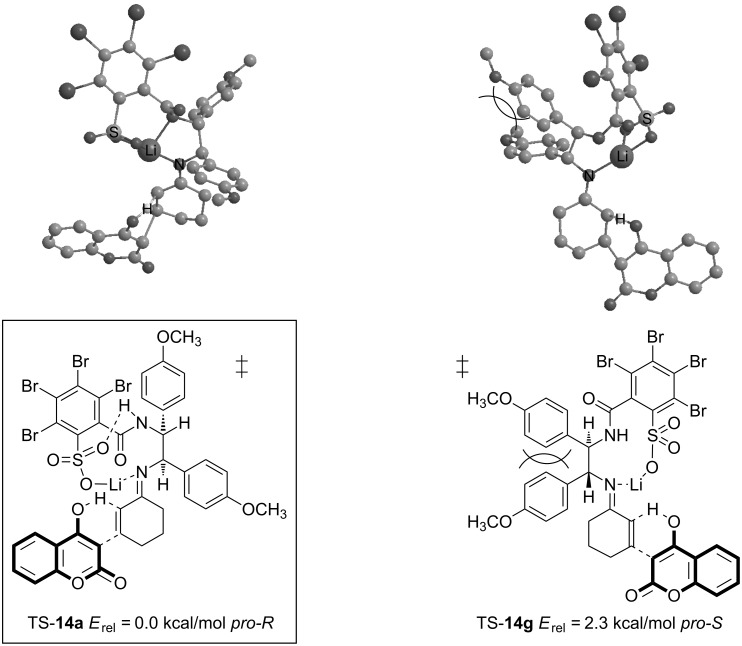
The competing enantio-determining transition structures TS-**14a** and TS-**14g**. The reason for the destabilization of **14g** (most stable *pro-S*) is the steric repulsion of anisyl groups in the backbone (TPSS/SVP+ZPE).

The transition structures shown in Figure 3 were initially calculated with the BP86 functional (relative energies in parenthesis). In order to ensure the results and increase the accuracy, structures **14a**–**h** were re-optimized employing the nonempirical TPSS functional. Relative energies in Figure 3 suggest that transition structure TS-**14a** is the most favored by 2.3 kcal/mol compared to the lowest competing TS in *pro-S*-configuration, TS-**14g**. Since TS-**14a** exhibits *pro-R-*configuration, the (*R*)-enantiomer of **17** should be generated in majority. The experimentally favored enantiomer is indeed (*R*)-configured with 83% ee (Table 2, entry 7). This value is in good agreement with an energetic differentiation of the enantio-determining transition states of 2.3 kcal/mol although no solvent dependent effects are taken into account [33]. (Solvent corrections to the single-point energies lead to similar results, which are given in Supporting Information File 1). An interesting detail concerning the conformational degrees of freedom in transition structures TS-**14** is the more favored alignment of the 2-sulfobenzoic moiety according to TS-**14a**–**d** (Figure 3 and Figure 4). This conformation allows the formation of an intramolecular bond between the lithium ion and the nitrogen of the amide group. A similar structural property is found in the X-ray crystallographic structure of compound **5** (Figure 1). The same conformation of the 2-sulfobenzoic moiety is fixed by two intramolecular hydrogen bonds. There is also a conspicuous reason for the relatively high energies of the transition states **14e**–**h**: anisyl groups in TS-**14e**–**h** are forced into a disfavored *syn*-conformation, which leads to steric repulsion (Figure 4).

## Conclusion

Four chiral enantiopure precatalysts based on chiral *trans*-1,2-diamines have been developed. These precatalysts, **5**, **6**, **7** and **8**, exhibit a primary amino group, which is protonated by a neighboring sulfonic acid group. The formed zwitterionic salts can be easily transformed to alkali-metal salts, which are able to form imine complexes with prochiral α,β-unsaturated ketones, which could be detected by ESIMS spectrometry in an exemplary case. It was found that the Lewis acidities of the alkali metal ions are strong enough to activate the imine homologous Michael systems for nucleophilic addition of 4-hydroxycoumarin in an efficient way since conversions are often in the quantitative range. Furthermore, the catalysts provide very mild pH conditions, since the pH values are in the range of 9. This is very unusual, since ordinary primary amine catalysts require strong acids as additives, which can destroy sensitive components [18]. Attempts to employ the metal-free zwitterionic salts did not succeed for this type of catalyst. The enantiomeric excesses that could be achieved by the use of **5**–**8** were moderate to good up to 83%, and the origin of chiral induction could be rationalized by means of DFT calculations. It was found that there is one conformation of the enantio-determining transition structure TS-**14** that dominates sufficiently because steric repulsion of the anisyl groups in the ligand backbone is minimized.

## Experimental

### General

Solvents used in chemical conversions were dried by standard methods and distilled under argon prior to use. The enantiomeric excesses of the chiral 4-hydroxycumarin derivatives were determined by chiral HPLC. Unless otherwise specified, we employed the La Chrome elite unit from Hitachi together with the chiral column Chiracel AD-H in 25 cm length. The flow was adjusted to 0.8 mL/min, the pressure was 32 bar and the detected wavelength λ = 240 nm. The eluent consisted of 80% hexanes and 20% isopropanol. The enantiomers of the chiral compounds were identified by reference spectra of racemates and literature data [23].

**Synthesis of compound 5**. The 2-sulfobenzoic anhydride **4**, R^1–4^ = H (1.751 mmol, 322 mg) was dissolved in 15 mL THF. The mixture was cooled to 0 °C and (1*R*,2*R*)-1,2-diaminocyclohexane (1.751 mmol, 200 mg) was added in 5 mL THF. The solution was soon thickened by white precipitate, and the suspension was stirred for 24 h. The solvent was removed in vacuum, and the white solid was purified by crystallization from methanol. Yield: 434 mg, crude, 83% and 307 mg, pure, 59%. Mp 344 °C (decomposition); IR (nujol) ν: 2970, 1665, 1596, 1251, 1141; ^1^H NMR (300 MHz, D_2_O) 0.88–1.14 (4H, m), 1.47–1.56 (2H, m), 1.74–1.98 (2H, m), 2.35–2.54 (1H, m), 3.38–3.57 (1H, m), 7.29–7.42 (1H, m), 7.40–7.61 (2H, m), 7.74–7.91 (1H, m); ^13^C NMR (75.5 MHz, D_2_O) 24.25, 24.50, 31.21, 33.07, 53.53, 56.57, 127.12, 128.28, 130.13, 131.46, 133.79, 139.07, 174.24; HRMS–ESI (*m*/*z*): [M + Na]^+^ calcd for C_13_H_18_N_2_NaO_4_S, 321.0879; found, 321.0882; X-ray crystal data: C_13_H_18_N_2_O_4_S, M = 298.35 g/mol, space group: *P*2_1_2_1_2_1_, *a* = 8.4485(2) Å, *b* = 12.1533(4) Å, *c* = 13.6221(5) Å, *V* = 1398.68(8) Å^3^, *Z* = 4, ρ = 1.417 g/mL, *T* = 100(2) K, λ = 0.71073 Å, µ = 0.246 mm^−1^, reflections: 3055, observed: 2842 (I>2σ(I)), parameters refined: 197, R1 = 0.0259, (I>2σ(I)) wR2 = 0.0621, GOF = 1.081, all hydrogen atoms fixed in an ideal position, nitrogen-bonded hydrogen refined. CCDC 872757 contains the supplementary crystallographic data for this paper. These data can be obtained free of charge from The Cambridge Crystallographic Data Centre via http://www.ccdc.cam.ac.uk/data_request/cif.

**Synthesis of compound 6**. The *2*-sulfobenzoic anhydride **4**, R^1–4^ = Br (0.918 mmol, 459 mg) was dissolved in 7 mL THF. The mixture was cooled to 0 °C and (1*R*,2*R*)*-*1,2*-*diaminocyclohexane (0.918 mmol, 105 mg) was added in 5 mL THF. The solution was soon thickened by grey precipitate, and the suspension was stirred for 24 h. The solvent was removed in vacuum, and the grey solid was purified by crystallization from methanol. Yield: 510 mg, 90%. Mp 348 °C (decomposition); IR (nujol) ν: 2953, 2854, 1649, 1539, 1456, 1377, 1199, 1086; ^1^H NMR (300 MHz, DMSO-*d*_6_) 1.23–1.47 (4H, m), 1.60–1.80 (2H, m), 1.81–2.05 (2H, m), 2.73–2.81 (1H, m), 3.80–3.84 (1H, m), 8.02 (3H, s), 8.75 (1H, d, *J* = 8.8 Hz); ^13^C NMR (75.5 MHz, DMSO-*d*_6_) 23.71, 24.45, 29.70, 30.64, 50.09, 55.85, 124.26, 125.17, 130.47, 132.04, 138.42, 145.56, 166.52; HRMS–ESI (*m*/*z*): [M + Na]^+^ calcd for C_13_H_14_Br_4_N_2_NaO_4_S, 636.7259; found, 636.7267.

**Synthesis of compound 7**. The 2-sulfobenzoic anhydride **4**, R^1–4^ = H (1.102 mmol, 300 mg) was dissolved in 3 mL DCM. The mixture was cooled to 0 °C, and compound **3** (1.102 mmol, 300 mg) was added in a mixture of 3 mL DCM and 3 mL diethyl ether. The solution was soon clouded by white precipitate, and the suspension was stirred for 24 h. The solvent was removed in vacuum, and the white solid was purified by column chromatography on silica gel (ethanol/DCM 1:1). Yield: 377 mg, 75%. Mp 249 °C; IR (nujol) ν: 2970, 2886, 1462, 1377, 1246, 1177, 820; ^1^H NMR (300 MHz, CDCl_3_) 3.56 (3H, s), 3.68 (3H, s), 4.72–4.76 (1H, d, *J* = 10.7 Hz), 5.76–5.79 (1H, m), 6.29–6.32 (2H, d, *J* = 7.9Hz), 6.65–6.68 (2H, d, *J* = 8.3 Hz), 7.06–7.14 (5H, m), 7.16–7.37 (2H, m), 7.55–7.56 (1H, m), 8.78–8.80 (1H, d, *J* = 6.5Hz); ^13^C NMR (75.5 MHz, CDCl_3_) 54.93, 55.11, 56.06, 59.84, 113.79, 113.93, 125.55, 127.41, 128.55, 128.99, 129.49, 129.92, 129.89, 130.16, 133.83, 140.73, 158.94, 159.44, 169.08; HRMS–ESI (*m*/*z*): [M + Na]^+^ calcd for C_23_H_24_N_2_NaO_6_S, 479.1247; found, 479.1249.

**Synthesis of compound 8**. The 2-sulfobenzoic anhydride **4**, R^1–4^ = Br (0.918 mmol, 459 mg) was dissolved in a mixture of 3 mL DCM and 3 mL THF. The solution was cooled to 0 °C, and compound **3** (0.918 mmol, 250 mg) was added in a mixture of 3 mL DCM and 3 mL THF. The solution was soon clouded by grey precipitate, and the suspension was stirred for 24 h. The solvent was removed in vacuum, and the grey solid was purified by column chromatography on silica gel (ethanol/DCM 1:1). Yield: 267 mg, 38%. Mp 304 °C (decomposition); IR (nujol) ν: 2968, 2845, 2841, 1643, 1614, 1456, 1377, 1246, 1039; ^1^H NMR (300 MHz, DMSO-*d*_6_) 3.64 (3H, s), 3.68 (3H, s), 4.50–4.53 (1H, d, *J* = 10.9 Hz), 5.41–5.47 (1H, t, *J* = 9.9 Hz), 6.68–6.71 (2H, d, *J* = 8.2 Hz), 6.79–6.82 (2H, d, *J* = 8.3 Hz), 7.14–7.17 (2H, d, *J* = 8.3Hz), 7.32–7.34 (2H, d, *J* = 8.3 Hz), 8.48 (3H, s), 9.09–9.12 (1H, d, *J* = 8.8 Hz); ^13^C NMR (75.5 MHz, DMSO-*d*_6_) 55.29, 55.51, 56.48, 59.96, 113.79, 114.21, 124.19, 125.21, 126.84, 129.36, 129.89, 130.42, 138.27, 145.59, 158.70, 159.67, 166.34; HRMS–ESI (*m*/*z*): [M + Na]^+^ calcd for C_13_H_18_N_2_NaO_4_S, 794.7627; found, 794.7630.

**Addition of 4-hydroxycoumarin to α,β-unsaturated ketones.** The employed ligand **5**–**8** (0.02 mmol) was dissolved in a 0.1 M solution of alkali metal hydroxide in methanol (0.2 mL). The solution was evaporated, and the residue was dissolved in 0.5 mL solvent. α,β-Unsaturated ketone (0.14 mmol) was added subsequently, and the solution was stirred for 10 min at room temperature and cooled to the desired reaction temperature prior to the addition of 4-hydroxycoumarin. 4-Hydroxycumarin (0.1 mmol, 16 mg) was added, and the conversion was monitored by TLC. The mixture was subjected to column chromatography without quenching, and all yields were determined as isolated yields. The ee’s were established by chiral HPLC according to racemic standards and literature data [23].

4-Hydroxy-3-(3-oxo-1-phenylbutyl)coumarin (warfarin). ^1^H and ^13^C NMR data were found to be in agreement with the literature data [23]. Chiral HPLC: The measurements were performed with an eluent consisting of 80% hexanes and 20% isopropanol on a Diacel Chiralpak AD-H column with 0.8 mL/min flow. The detector wavelength was 254 nm. *t*_R_ = 10.1 and 25.5 min.

4-Hydroxy-3-(3-oxocyclohexyl)coumarin. ^1^H and ^13^C NMR data were found to be in agreement with the literature data [23]. Chiral HPLC: The measurements were performed with an eluent consisting of 80% hexanes and 20% isopropanol on a Diacel Chiralpak AD-H column with 0.8 mL/min flow. The detector wavelength was 254 nm. *t*_R_ = 12.0 min (minor, (*S*)-enantiomer) and 13.3 min (major, (*R*)-enantiomer [23].

4-Hydroxy-3-(3-oxocycloheptyl)coumarin. ^1^H and ^13^C NMR data were found to be in agreement with the literature data [23]. Chiral HPLC: The measurements were performed with an eluent consisting of 90% hexanes and 10% isopropanol on a Diacel Chiralpak AS-H column with 1.0 mL/min flow. The detector wavelength was 210 nm. *t*_R_ = 22.5 and 31.4 min.

**pH Measurements.** The pH-range of the catalytic system was determined for the precatalyst **5** with lithium as Lewis acid. Conditions were chosen according to Table 1, entry 3, and the samples were prepared according to the procedure of catalysis. The solution of Li-**5** in the specified solvent mixture was tested with wet universal pH indicator strips from Merck to give a pH of 9. After the addition of benzylideneacetone and stirring for 15 min at room temperature, the pH value was measured again to be 8–9. All measurements were repeated three times and three samples were prepared to give identical results.

**Computational details.** All theoretical calculations were performed with the program package TURBOMOLE-6.3 [34]. The employed density functionals were the nonempirical TPSS-functional developed by Tao, Perdew, Scuseria and Staroverov [35] or the BP86-functional (as specified), combined with the contracted SVP basis set from Aldrich et al. [36]. The multipole accelerated resolution of identity approximation for two electron integral evaluation was used. All stationary points were fully optimized and confirmed by separate analytical frequency calculations. Transition structures were optimized with quasi-Newton-Raphson methods by using the Powell-update algorithm for hessian matrix approximation (subsequent analytical frequency calculation). Absolute energies were zero-point corrected with the vibrational information received from harmonic analytical frequency calculations.

## Supporting Information

File 1 Spectra of precatalysts and crystallographic data for compound **5**.
