# Supplementary material for: Metal-mediated aminocatalysis provides mild conditions: Enantioselective Michael addition mediated by primary amino catalysts and alkali-metal ions
Source: Beilstein J Org Chem. 2013 Jan 23;9:155–65. doi: 10.3762/bjoc.9.18 (PMC3566766; doi:10.3762/bjoc.9.18)
Supplement: File 1 — Spectra of precatalysts and crystallographic data for compound 5. [file Beilstein_J_Org_Chem-09-155-s001.pdf]

Supporting Information
for

Metal-mediated aminocatalysis provides mild conditions: Enantioselective Michael addition mediated by primary amino catalysts and alkali-metal ions

Matthias Leven, Jörg M. Neudörfl[†], and Bernd Goldfuss*

Address: Department of Chemistry, Universität zu Köln, Greinstrasse 4, D-50939 Köln, Germany, Fax: +49(0)221-470-5057

Email: Bernd Goldfuss - goldfuss@uni-koeln.de

*Corresponding author

[†]X-ray analysis

Spectra of precatalysts and crystallographic data for compound 5.

NMR spectroscopy

General: Deuterated solvents where purchased from Deutero GmbH, Kastellaun (Germany) (chloroform-d and DMSO-d_6) and Sigma Aldrich (D_2O). Chloroform-d was dried over molecular sieves. NMR spectra for characterization of compounds where recorded with a Bruker DPX 300 spectrometer (^{1}H frequency 300.13 MHz).

^{1}H -and ^{13}C-NMR spectra of compound **5** (D_2O):

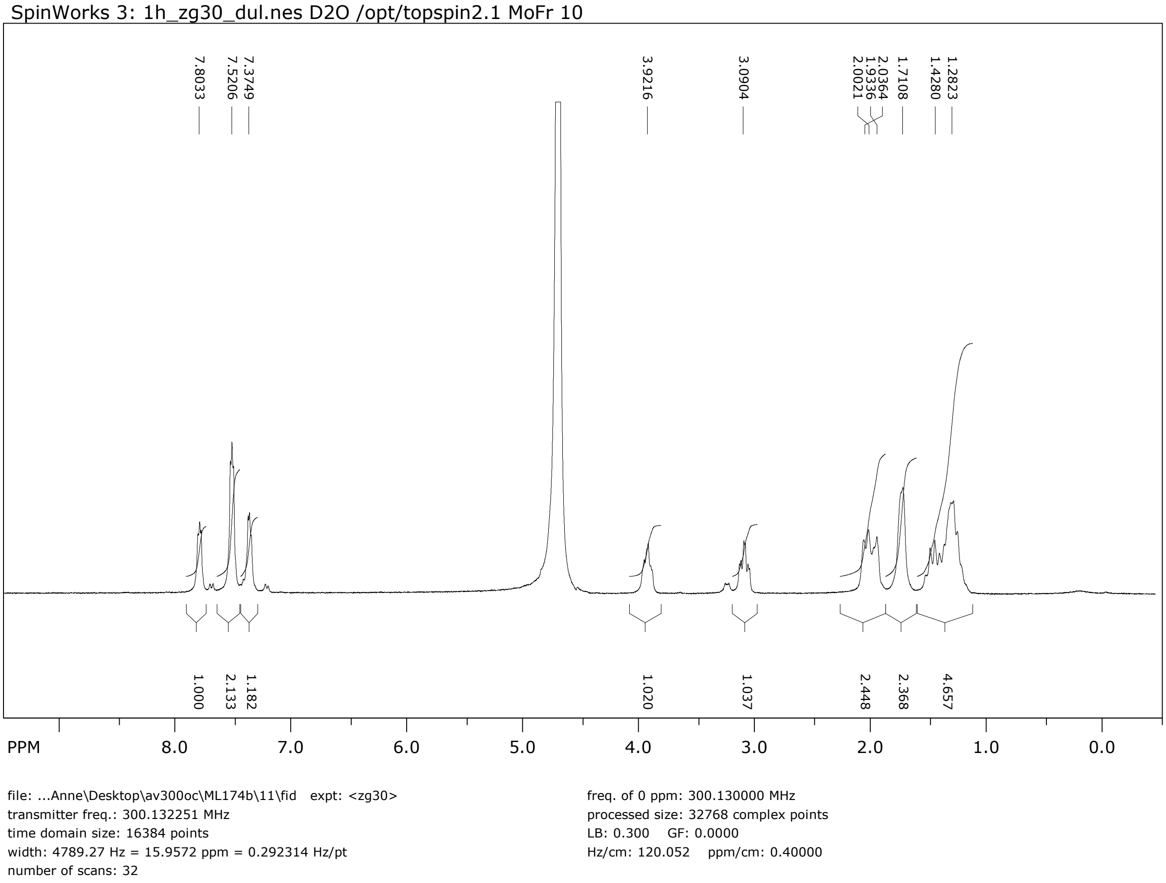

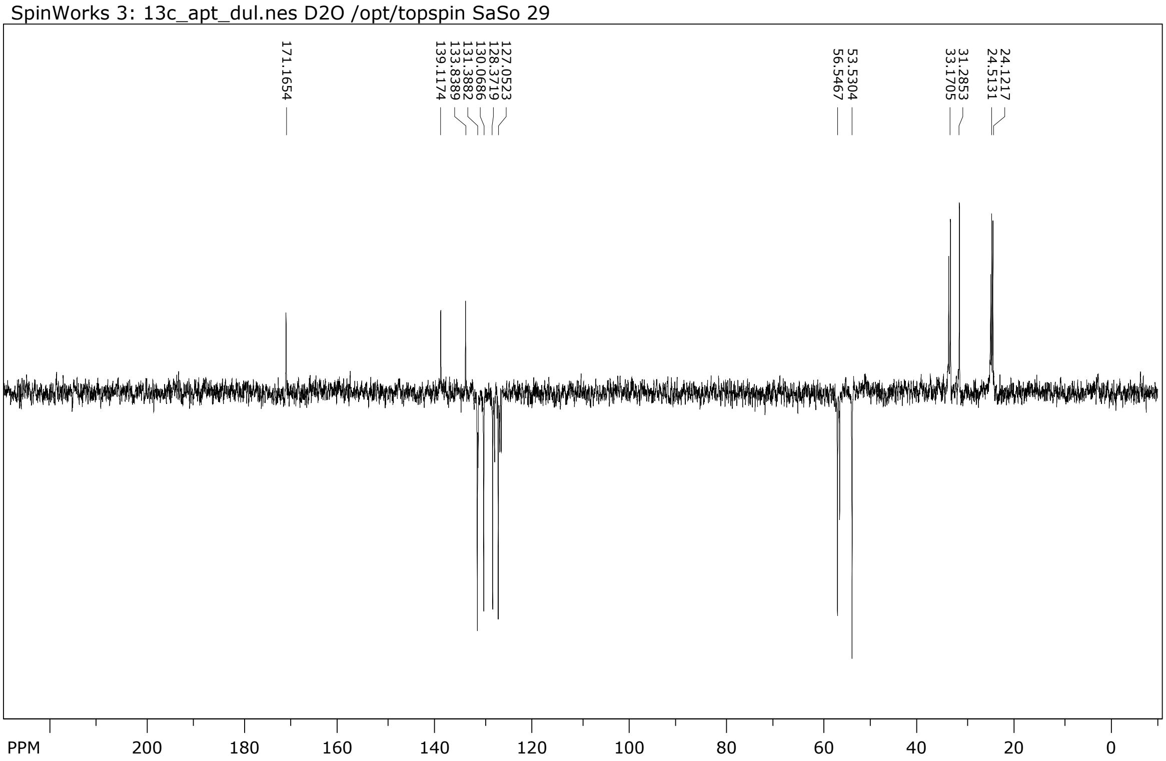

file: ...\dpx300\ML169Dach_amid_so3h\10\fid expt: <apt>
transmitter freq.: 75.476050 MHz
time domain size: 32768 points
width: 18115.94 Hz = 240.0224 ppm = 0.552855 Hz/pt
number of scans: 512

freq. of 0 ppm: 75.467749 MHz
processed size: 32768 complex points
LB: 3.000 GF: 0.0000
Hz/cm: 724.638 ppm/cm: 9.60090

^{1}H and ^{13}C NMR spectra of compound **6** (DMSO-d_6):

SpinWorks 3: 13c_apt_dul.nes DMSO /opt/topspin MoFr 5

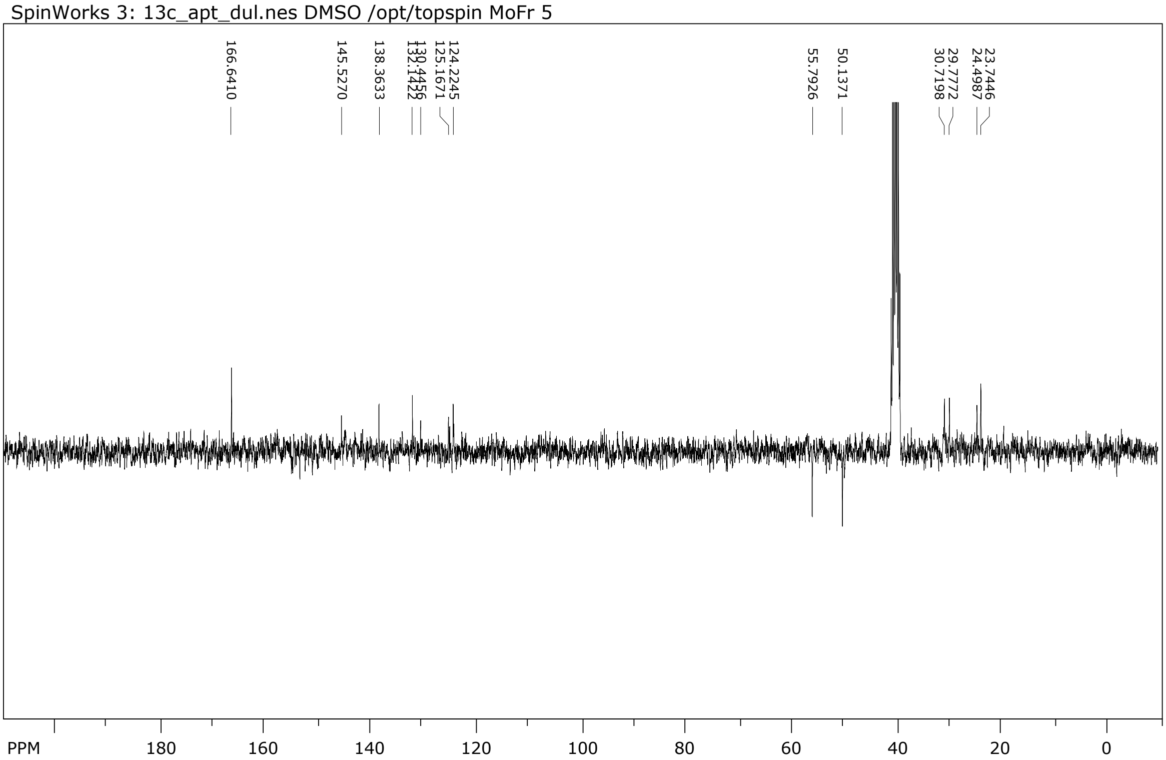

file: ...300\ML212b_Br4_Dach_SO3H_uk\10\fid expt: <apt>
transmitter freq.: 75.476050 MHz
time domain size: 32768 points
width: 18115.94 Hz = 240.0224 ppm = 0.552855 Hz/pt
number of scans: 512

freq. of 0 ppm: 75.467749 MHz
processed size: 32768 complex points
LB: 3.000 GF: 0.0000
Hz/cm: 664.116 ppm/cm: 8.79903

SpinWorks 3: 1h_zg30_dul.nes DMSO /opt/topspin MoFr 5

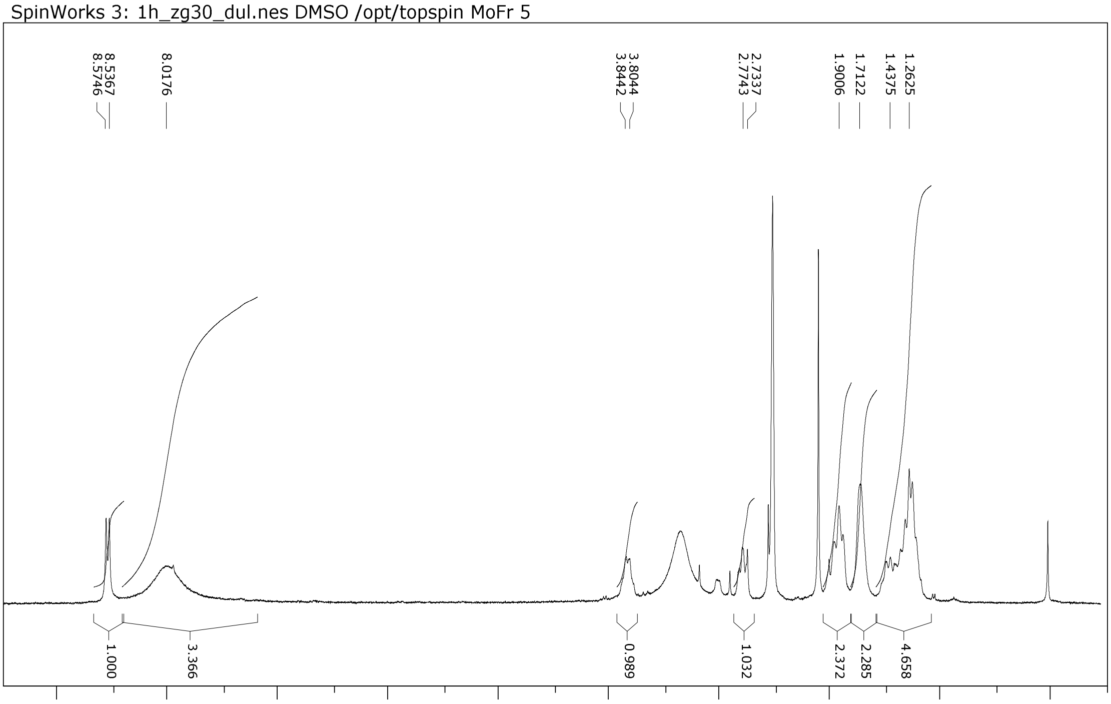

file: ...300\ML212b_Br4_Dach_SO3H_uk\11\fid expt: <zg30>
transmitter freq.: 300.132251 MHz
time domain size: 16384 points
width: 4789.27 Hz = 15.9572 ppm = 0.292314 Hz/pt
number of scans: 32

freq. of 0 ppm: 300.130000 MHz
processed size: 32768 complex points
LB: 0.300 GF: 0.0000
Hz/cm: 120.052 ppm/cm: 0.40000

^{1}H and ^{13}C NMR spectra of compound **7** ($CDCl_3$):

SpinWorks 3: 1h_zg30_dul.nes CDCl3 /opt/topspin2.1 MoFr 5

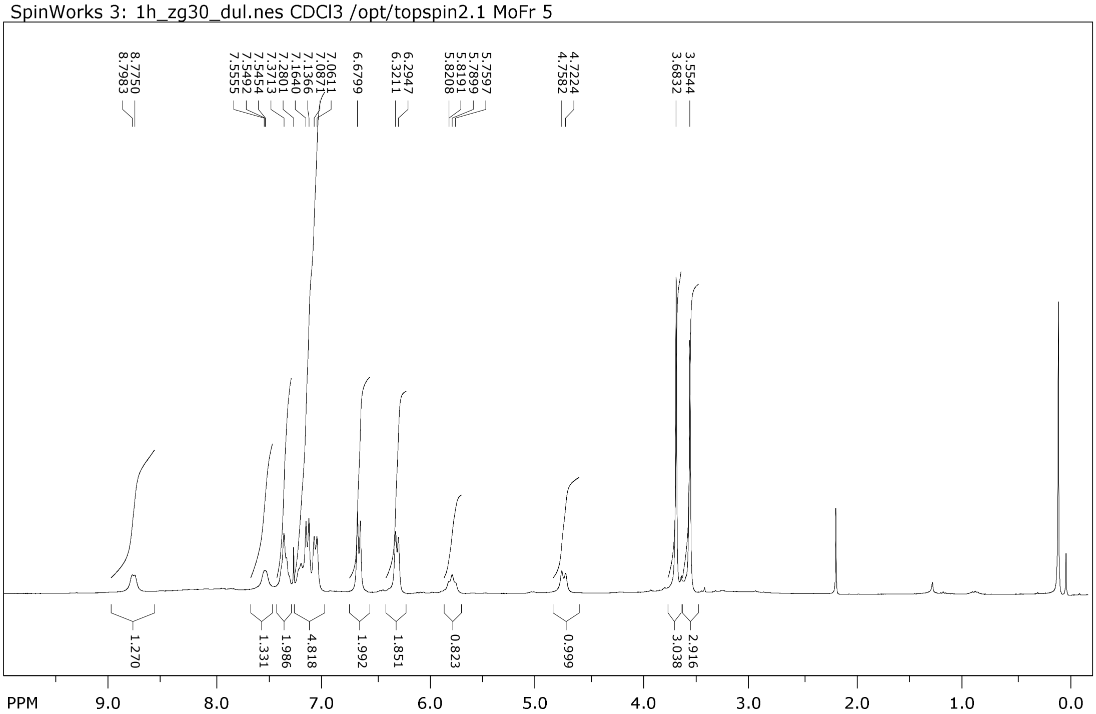

file: ...Anne\Desktop\av300oc\ML174c\11\fid expt: <zg30>
transmitter freq.: 300.132251 MHz
time domain size: 16384 points
width: 4789.27 Hz = 15.9572 ppm = 0.292314 Hz/pt
number of scans: 32

freq. of 0 ppm: 300.130000 MHz
processed size: 32768 complex points
LB: 0.300 GF: 0.0000
Hz/cm: 122.461 ppm/cm: 0.40802

SpinWorks 3: 13c_deptqgp_dul CDCl3 /opt/topspin2.1 MoFr 5

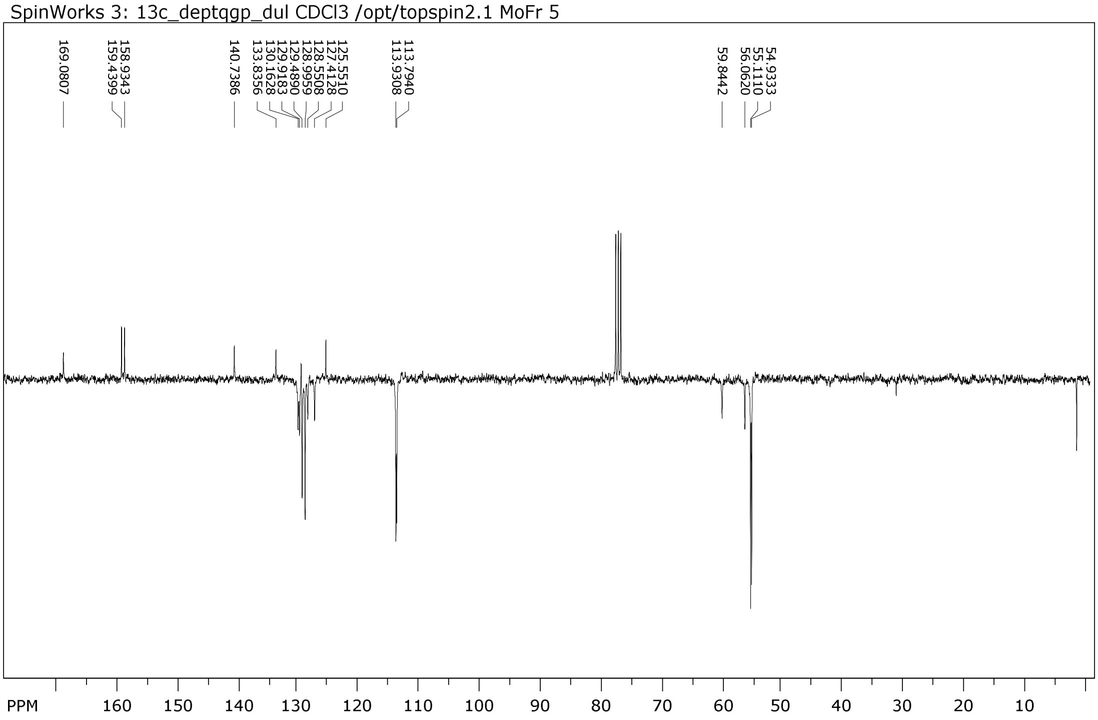

file: ...Anne\Desktop\av300oc\ML174c\10\fid expt: <deptqgpsp>
transmitter freq.: 75.476050 MHz
time domain size: 32768 points
width: 18115.94 Hz = 240.0224 ppm = 0.552855 Hz/pt
number of scans: 360

freq. of 0 ppm: 75.467749 MHz
processed size: 32768 complex points
LB: 3.000 GF: 0.0000
Hz/cm: 543.944 ppm/cm: 7.20684

^{1}H and ^{13}C NMR spectra of compound **8** (DMSO-d$_6$):

SpinWorks 3: 13c_apt_dul.nes DMSO /opt/topspin MoFr 1

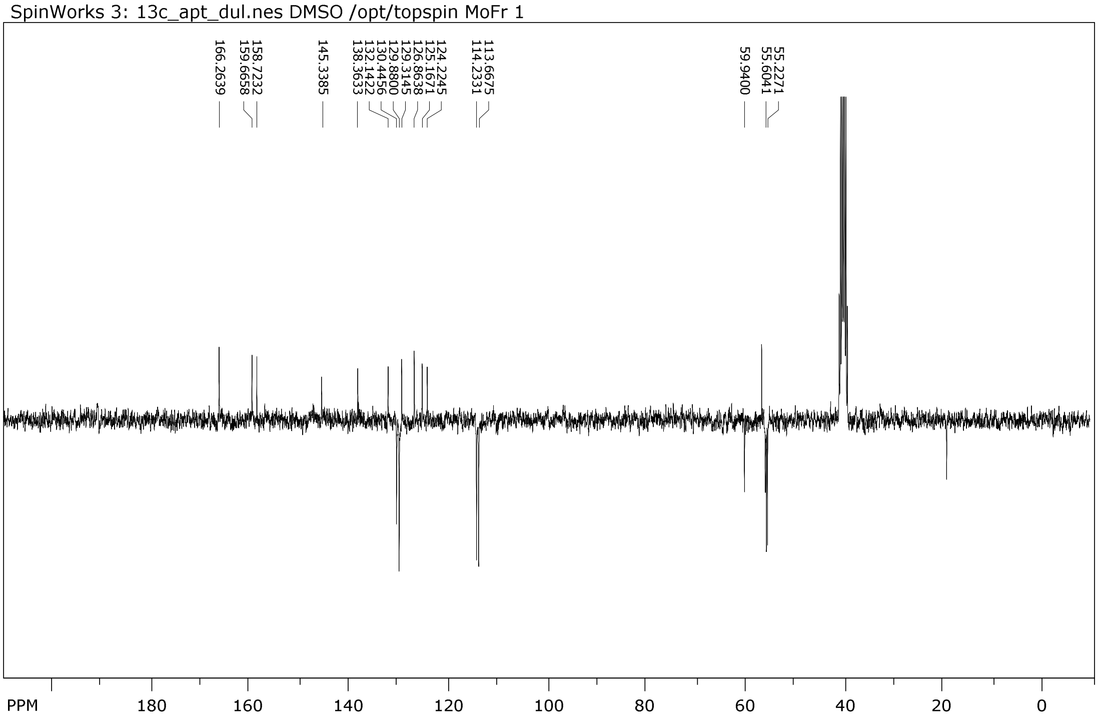

file: ...n\Anne\Desktop\dpx300\ML210\10\fid expt: <apt>
transmitter freq.: 75.476050 MHz
time domain size: 32768 points
width: 18115.94 Hz = 240.0224 ppm = 0.552855 Hz/pt
number of scans: 512

freq. of 0 ppm: 75.467749 MHz
processed size: 32768 complex points
LB: 3.000 GF: 0.0000
Hz/cm: 664.116 ppm/cm: 8.79903

SpinWorks 3: 1h_zg30_dul.nes DMSO /opt/topspin MoFr 1

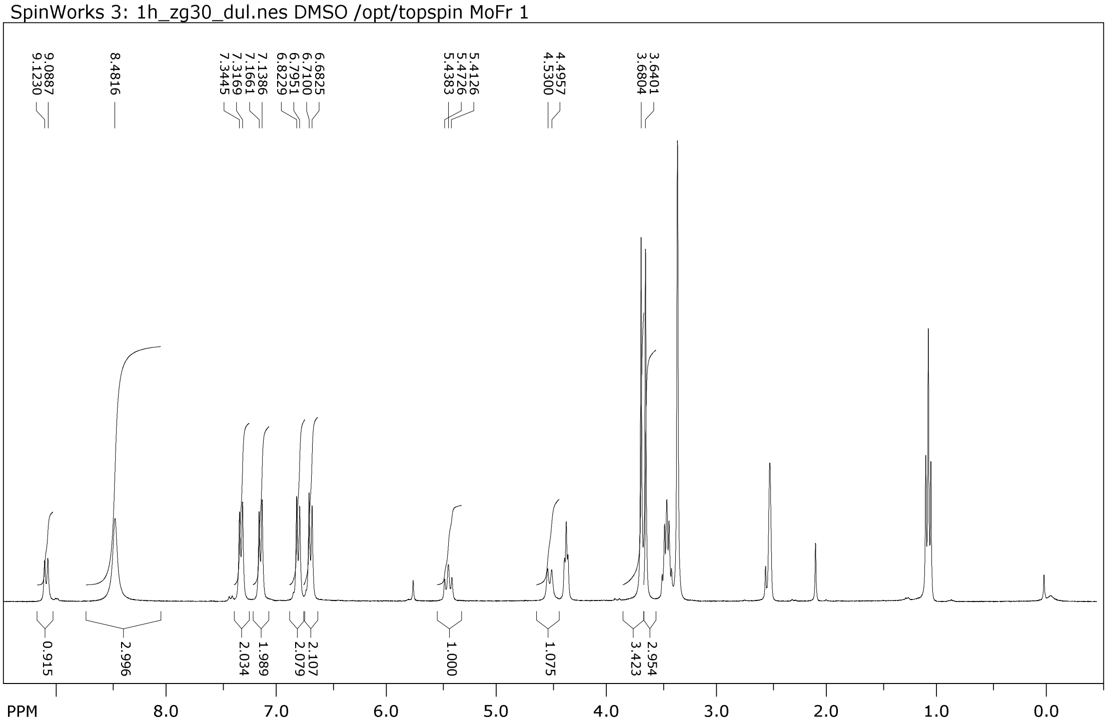

file: ...n\Anne\Desktop\dpx300\ML210\11\fid expt: <zg30>
transmitter freq.: 300.132251 MHz
time domain size: 16384 points
width: 4789.27 Hz = 15.9572 ppm = 0.292314 Hz/pt
number of scans: 32

freq. of 0 ppm: 300.130000 MHz
processed size: 32768 complex points
LB: 0.300 GF: 0.0000
Hz/cm: 120.052 ppm/cm: 0.40000

4-hydroxy-3-(3-oxo-1-phenylbutyl)cumarin (warfarin/$CDCl_3$)

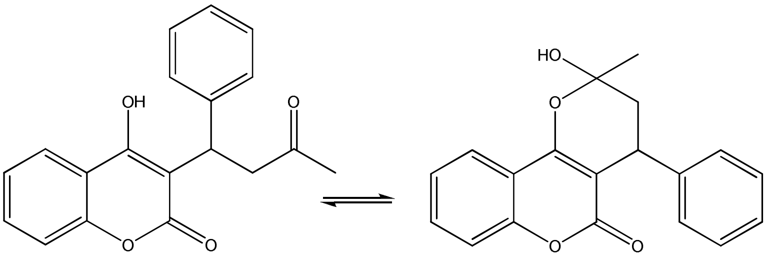

SpinWorks 3: 13c_apt_dul.nes CDCl3 /opt/topspin SaSo 16

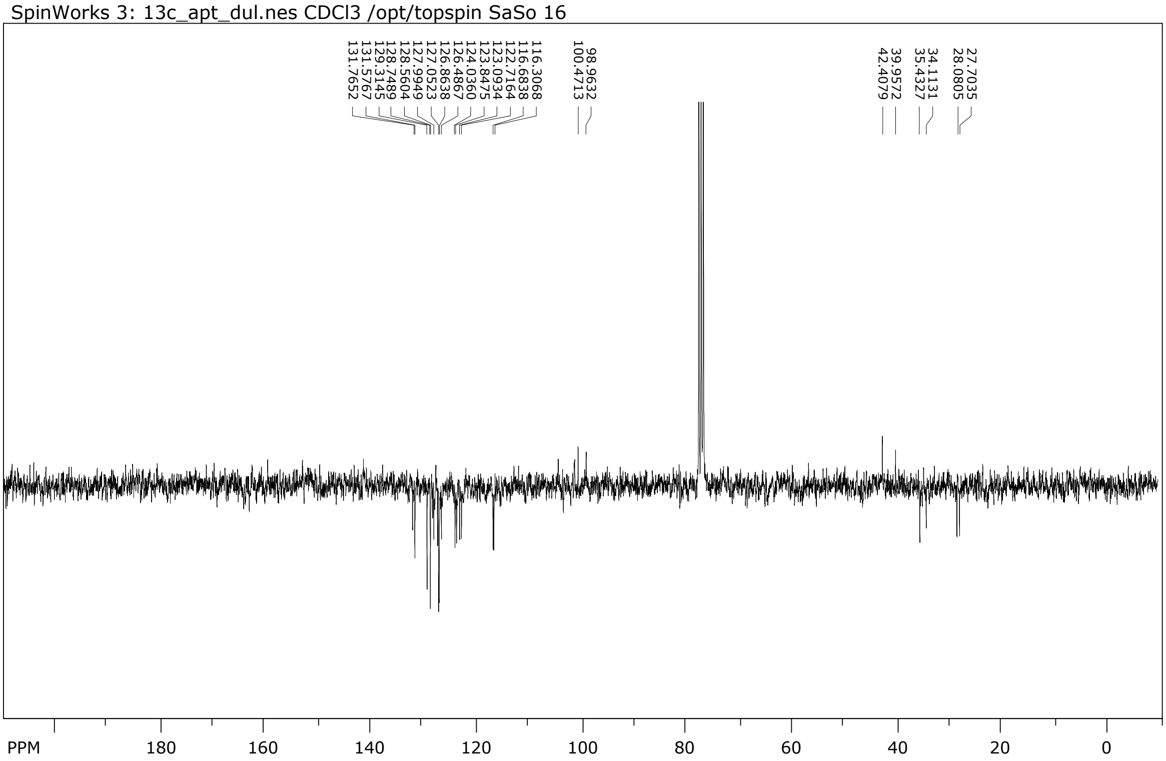

file: ...n\Anne\Desktop\dpx300\ML198\10\fid expt: <apt>
transmitter freq.: 75.476050 MHz
time domain size: 32768 points
width: 18115.94 Hz = 240.0224 ppm = 0.552855 Hz/pt
number of scans: 512

freq. of 0 ppm: 75.467749 MHz
processed size: 32768 complex points
LB: 3.000 GF: 0.0000
Hz/cm: 664.116 ppm/cm: 8.79903

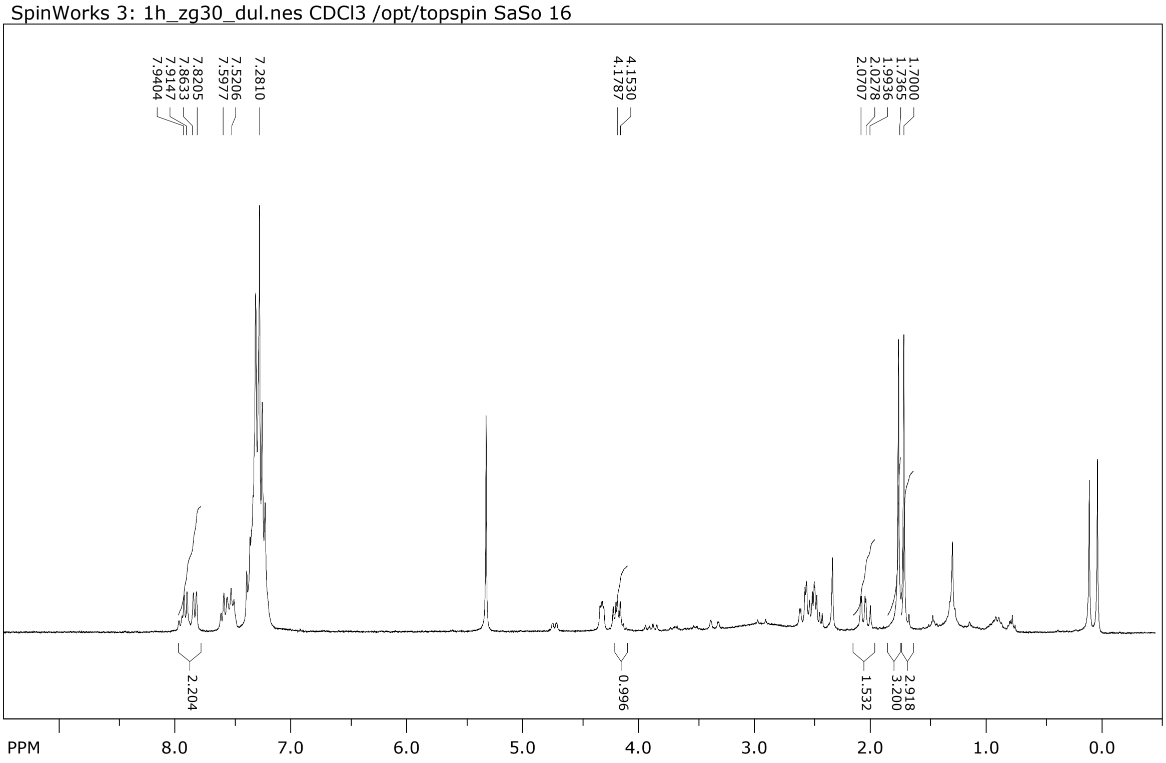

file: ...n\Anne\Desktop\dpx300\ML198\11\fid expt: <zg30>
transmitter freq.: 300.132251 MHz
time domain size: 16384 points
width: 4789.27 Hz = 15.9572 ppm = 0.292314 Hz/pt
number of scans: 32

freq. of 0 ppm: 300.130000 MHz
processed size: 32768 complex points
LB: 0.300 GF: 0.0000
Hz/cm: 120.052 ppm/cm: 0.40000

4-hydroxy-3-(3-oxocyclohexyl)cumarin ($CDCl_3$)

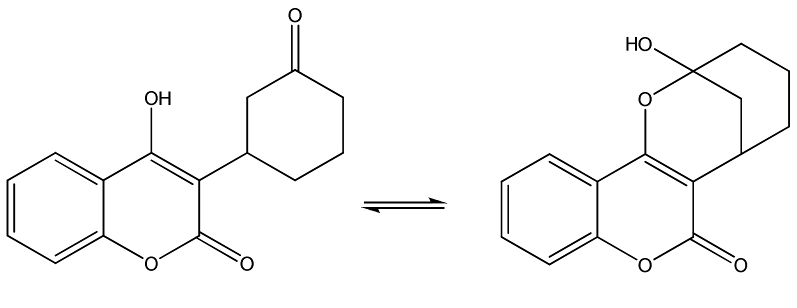

SpinWorks 3: 13c_apt_dul.nes CDCl3 /opt/topspin MoFr 2

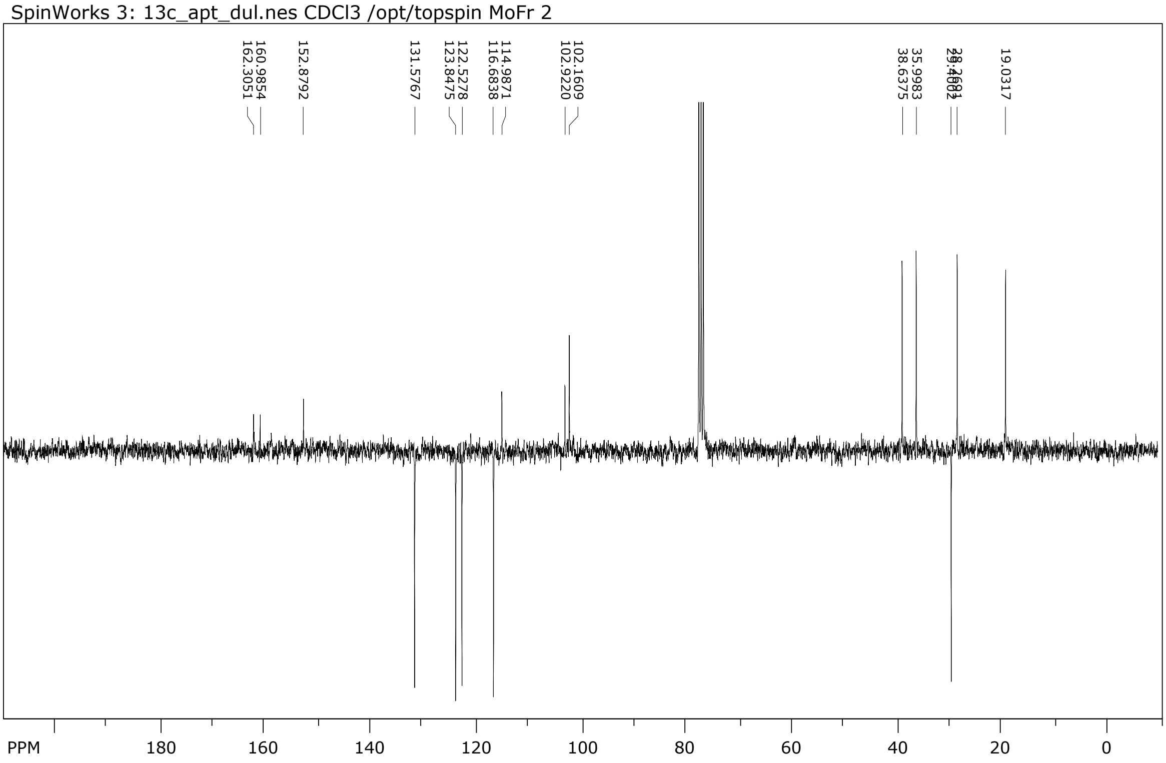

file: ...n\Anne\Desktop\dpx300\ML202\10\fid expt: <apt>
transmitter freq.: 75.476050 MHz
time domain size: 32768 points
width: 18115.94 Hz = 240.0224 ppm = 0.552855 Hz/pt
number of scans: 512

freq. of 0 ppm: 75.467749 MHz
processed size: 32768 complex points
LB: 3.000 GF: 0.0000
Hz/cm: 664.116 ppm/cm: 8.79903

SpinWorks 3: 1h_zg30_dul.nes CDCl3 /opt/topspin MoFr 2

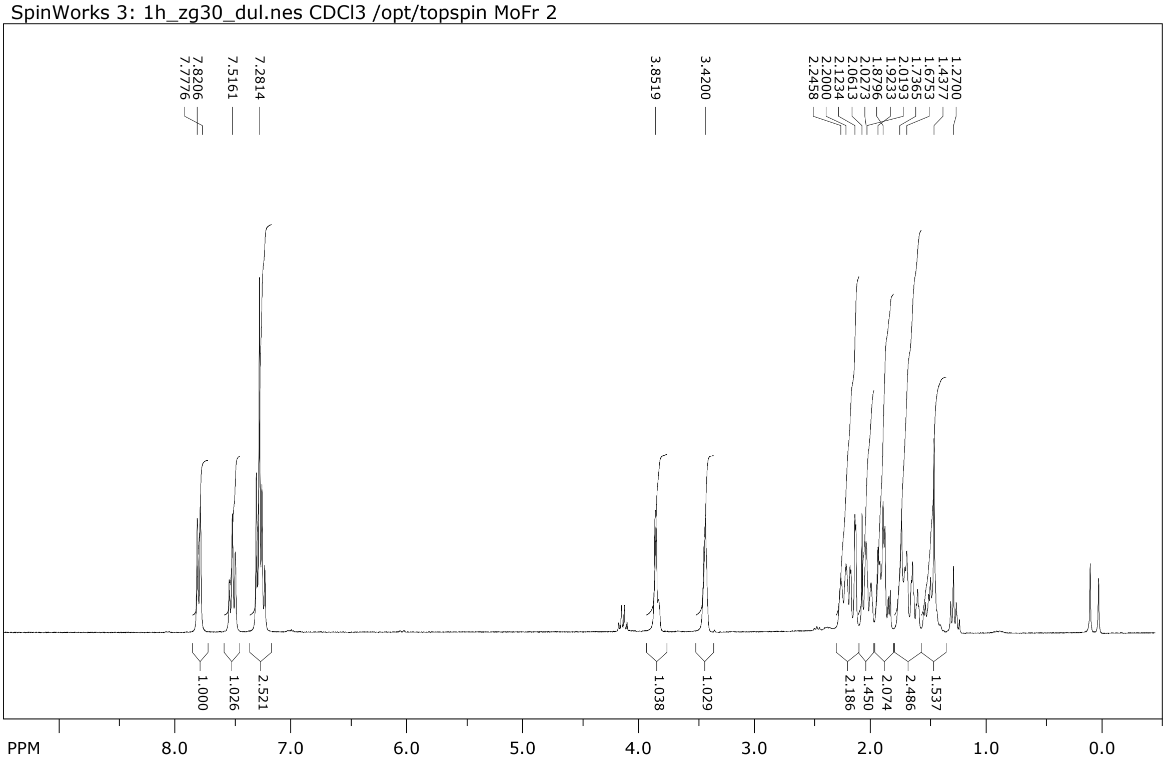

file: ...n\Anne\Desktop\dpx300\ML202\11\fid expt: <zg30>
transmitter freq.: 300.132251 MHz
time domain size: 16384 points
width: 4789.27 Hz = 15.9572 ppm = 0.292314 Hz/pt
number of scans: 32

freq. of 0 ppm: 300.130000 MHz
processed size: 32768 complex points
LB: 0.300 GF: 0.0000
Hz/cm: 120.052 ppm/cm: 0.40000

4-hydroxy-3-(3-oxocycloheptyl)cumarin ($CDCl_3$)

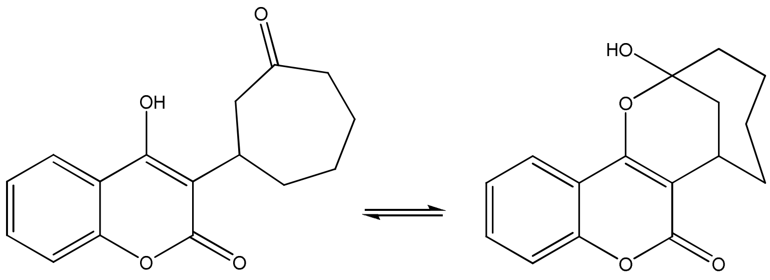

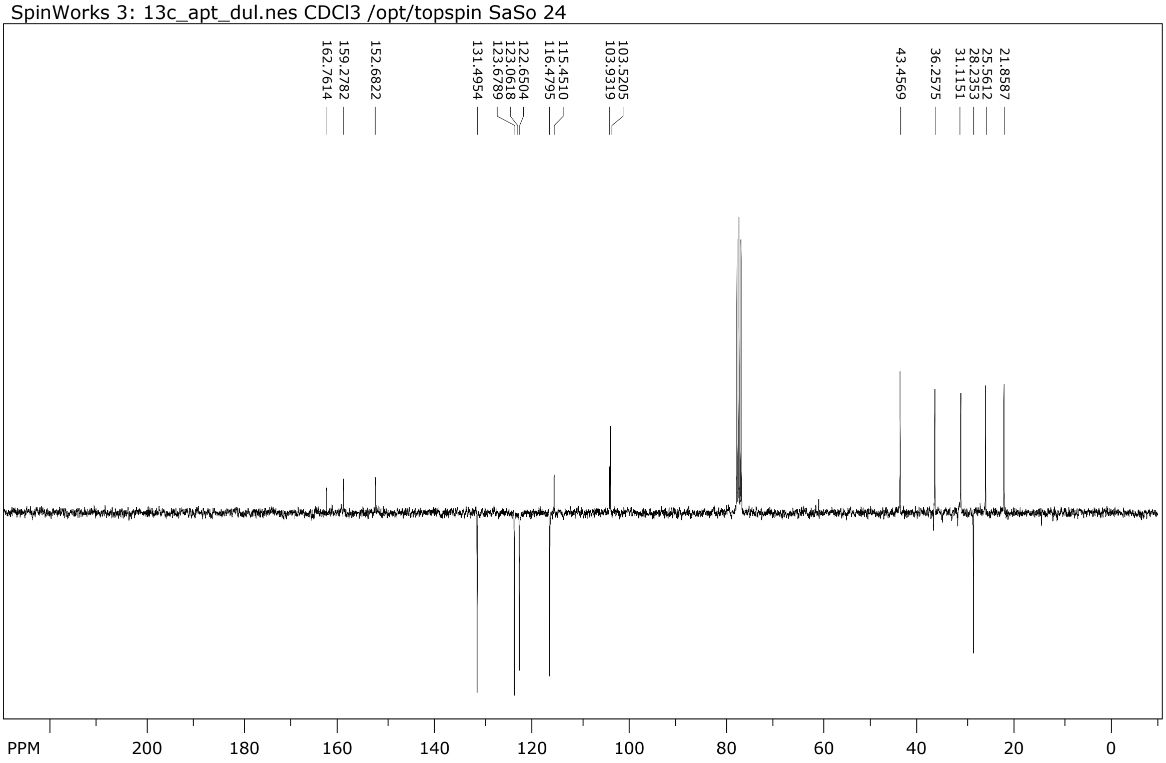

file: ...n\Anne\Desktop\dpx300\ML214\10\fid expt: <apt>
transmitter freq.: 75.476050 MHz
time domain size: 32768 points
width: 18115.94 Hz = 240.0224 ppm = 0.552855 Hz/pt
number of scans: 512

freq. of 0 ppm: 75.467749 MHz
processed size: 32768 complex points
LB: 3.000 GF: 0.0000
Hz/cm: 724.638 ppm/cm: 9.60090

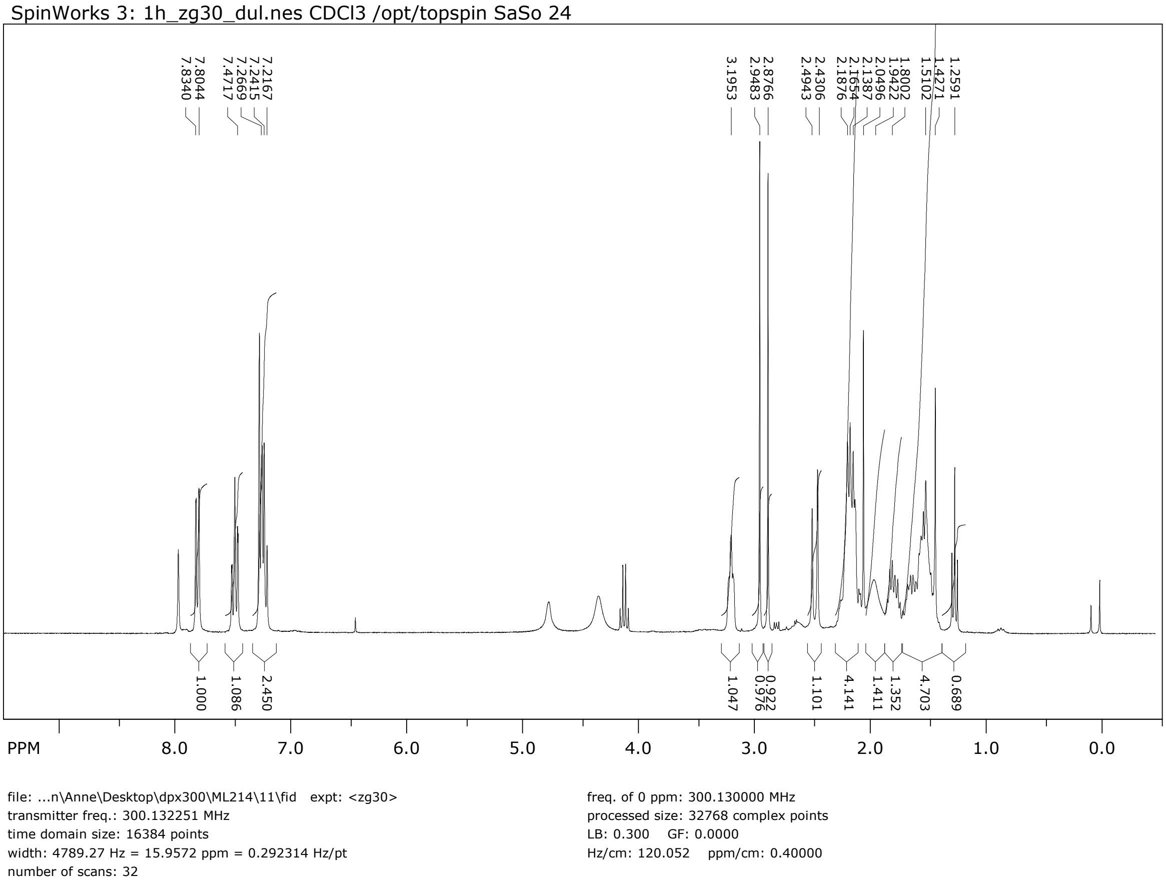

ESI-MS

High-resolution MS was carried out with ESI-LC/MS in positive mode with a resolution of 30.000.

ESI-MS measurements for detection of intermediates from the catalytic process were carried out in positive mode employing a Bruker Daltonics Esquire 3000 plus. The flow rate was adjusted at 9µl/min and HPLC grade methanol was used as eluent. Samples were prepared as follows: 0.02 mmol of compound **8**, were dissolved in a 0.1 M solution of alkali-metal hydroxide in methanol, 0.2 ml. The solution was evaporated and the residue was dissolved in 0.5 ml absolute THF. 0.14 mmol cyclohexenon was added subsequently and the solution was stirred for 10 min at room temperature prior to measurements.

Acquisition Parameter

Ion Source Type	ESI	Ion Polarity	Positive	Alternating Ion Polarity	off
Mass Range Mode	Std/Normal	Scan Begin	100 m/z	Scan End	2800 m/z
Capillary Exit	220.0 Volt	Skim 1	40.0 Volt	Trap Drive	78.0
Accumulation Time	624 µs	Averages	7 Spectra	Auto MS/MS	off

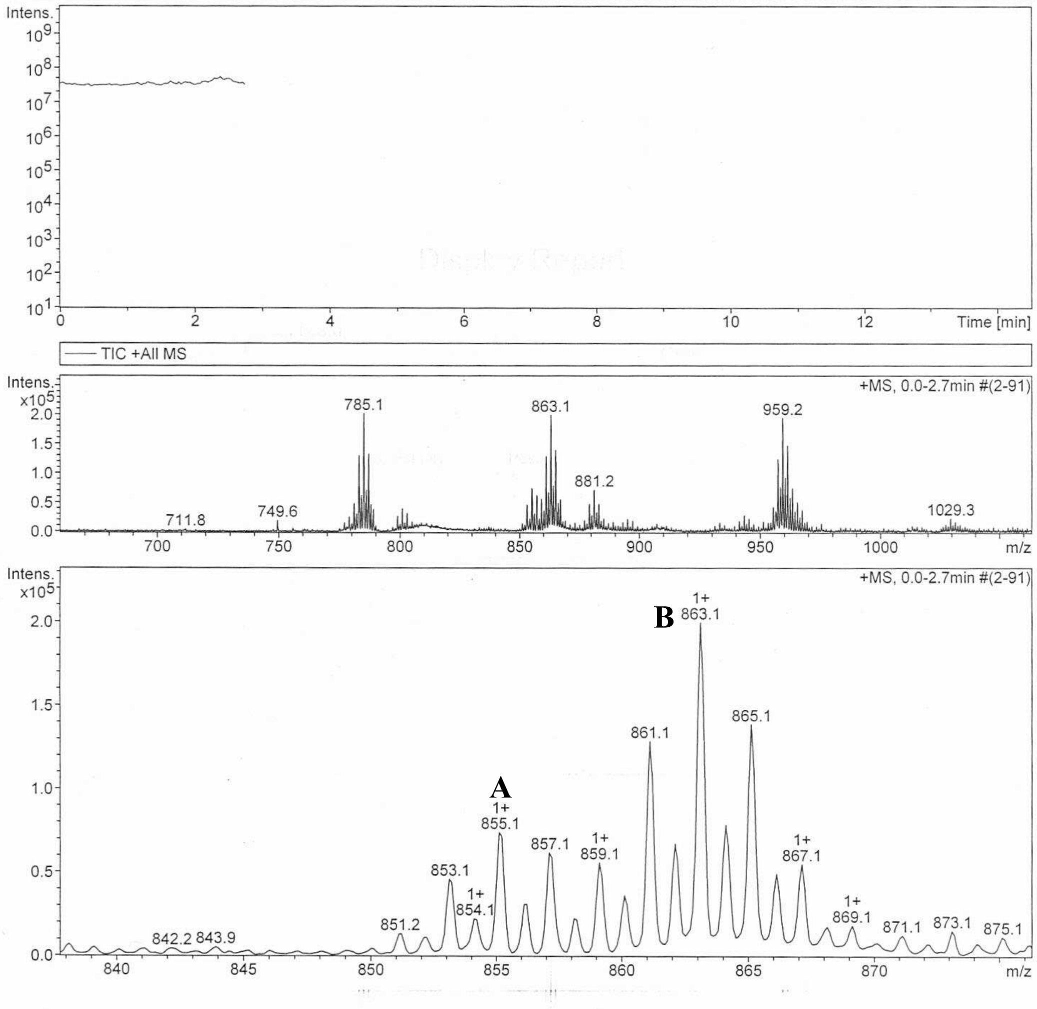

Bruker Daltonics DataAnalysis 3.3 printed: 01.03.2012 19:17:18 Page 1 of 1

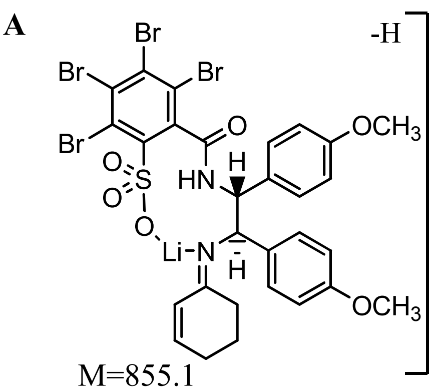

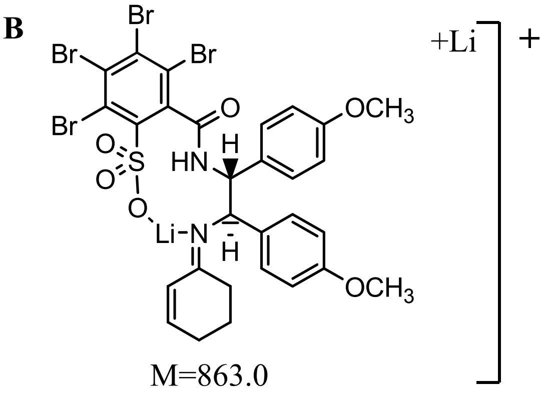

X-ray crystallography

Compound **5** was crystallized from methanol. The colourless crystals were used for X-ray crystallography subsequently.

```
TITL hk171 100k .3 .1 .1
CELL  0.71073    8.4485   12.1533   13.6221    90.000    90.000    90.000
ZERR  4           0.0002    0.0004    0.0005     0.000     0.000     0.000
LATT  -1
SYMM     0.50000 - X ,            - Y ,    0.50000 + Z
SYMM     0.50000 + X ,    0.50000 - Y ,            - Z
SYMM             - X ,    0.50000 + Y ,    0.50000 - Z
SFAC  C  H  N  O  S
UNIT    52    72   8  16   4
OMIT -2 54
TEMP -173
L.S. 5
FMAP 2
PLAN 20
ACTA
BOND $H
CONF
WGHT    0.038700
FVAR       0.38611
S1    5    0.381274    0.471094   -0.043850    11.00000    0.01696
0.01543 =
         0.01512   -0.00198    0.00171   -0.00101
O1    4    0.445155    0.535427   -0.123325    11.00000    0.03007
0.02089 =
         0.01913   -0.00136    0.00736   -0.00407
O2    4    0.256143    0.394442   -0.071901    11.00000    0.02066
0.01704 =
         0.01950   -0.00325   -0.00208   -0.00198
O3    4    0.504630    0.414400    0.013955    11.00000    0.01443
0.02145 =
         0.02342   -0.00198    0.00052    0.00278
O4    4    0.012752    0.379221    0.140492    11.00000    0.01478
0.01901 =
         0.02307    0.00047   -0.00016   -0.00231
N1    3    0.275801    0.347414    0.157103    11.00000    0.01380
0.01424 =
         0.01803    0.00090    0.00053   -0.00202
N2    3    0.301494    0.180813    0.004168    11.00000    0.01649
0.01447 =
         0.02400   -0.00184    0.00209   -0.00046
C1    1    0.290422    0.565405    0.038972    11.00000    0.01485
0.01743 =
         0.01469   -0.00087   -0.00216    0.00110
C2    1    0.189975    0.530536    0.115147    11.00000    0.01424
0.01900 =
         0.01364    0.00085   -0.00315    0.00077
C3    1    0.152109    0.412059    0.137452    11.00000    0.01689
0.01767 =
         0.01166   -0.00223    0.00075    0.00061
C4    1    0.268908    0.230022    0.179523    11.00000    0.01711
0.01322 =
         0.02183    0.00383   -0.00092   -0.00006
C5    1    0.366211    0.164974    0.104886    11.00000    0.01317
0.01608 =
         0.02545    0.00021   -0.00189   -0.00044
```

```
C6    1    0.367984    0.042368    0.129038    11.00000    0.01784
0.01455 =
         0.04155   -0.00090   -0.00397    0.00150
C7    1    0.426290    0.021746    0.232825    11.00000    0.02041
0.01885 =
         0.05227    0.01143   -0.01168    0.00129
C8    1    0.330330    0.085317    0.307798    11.00000    0.02765
0.02847 =
         0.03390    0.01400   -0.00850   -0.00321
C9    1    0.328798    0.208603    0.283500    11.00000    0.02834
0.02523 =
         0.02304    0.00508   -0.00438    0.00028
C10   1    0.119052    0.609313    0.175284    11.00000    0.01712
0.02027 =
         0.01925   -0.00038   -0.00003    0.00317
C11   1    0.146652    0.721039    0.160899    11.00000    0.02556
0.01917 =
         0.02257   -0.00471   -0.00024    0.00505
C12   1    0.245826    0.754252    0.085654    11.00000    0.02826
0.01494 =
         0.02682   -0.00058   -0.00248   -0.00047
C13   1    0.318034    0.677167    0.024778    11.00000    0.02023
0.01750 =
         0.01949    0.00041   -0.00008   -0.00101
H1    2    0.265650    0.014397    0.121651    11.00000    0.03178
H2    2    0.365202    0.150830   -0.044421    11.00000    0.03117
H3    2    0.155778    0.207954    0.171727    11.00000    0.02012
H4    2    0.267905    0.835318    0.070567    11.00000    0.03665
H5    2    0.050963    0.585577    0.228373    11.00000    0.00833
H6    2    0.437882    0.235491    0.288774    11.00000    0.02610
H7    2    0.469762    0.192553    0.107176    11.00000    0.01388
H8    2    0.376974    0.071987    0.374859    11.00000    0.03058
H9    2    0.195597    0.144695    0.003520    11.00000    0.03779
H10   2    0.357298    0.373453    0.149566    11.00000    0.01196
H11   2    0.394337    0.697031   -0.026867    11.00000    0.03629
H12   2    0.096165    0.775160    0.204267    11.00000    0.02324
H13   2    0.291331    0.254524   -0.009696    11.00000    0.03030
H14   2    0.425249    0.006254    0.086982    11.00000    0.02715
H15   2    0.259712    0.250737    0.330416    11.00000    0.02705
H16   2    0.531344    0.043217    0.239159    11.00000    0.04228
H17   2    0.216351    0.058613    0.307901    11.00000    0.03909
H18   2    0.417353   -0.055574    0.248174    11.00000    0.02751
HKLF 4

REM  hk171 100k .3 .1 .1
REM R1 =  0.0260 for   2842 Fo > 4sig(Fo)  and  0.0301 for all   3055 data
REM    253 parameters refined using      0 restraints

END

WGHT      0.0384      0.0000
REM Highest difference peak  0.160,  deepest hole -0.238,  1-sigma level
0.039
Q1    1   0.3711  0.1085  0.1260  11.00000  0.05    0.16
Q2    1   0.0936  0.6627  0.1559  11.00000  0.05    0.16
Q3    1   0.3167  0.1958  0.1444  11.00000  0.05    0.15
Q4    1   0.3567  0.5223  0.0129  11.00000  0.05    0.15
Q5    1   0.2368  0.5415  0.0789  11.00000  0.05    0.14
Q6    1   0.1687  0.4769  0.1261  11.00000  0.05    0.13
Q7    1   0.4770 -0.0023  0.0536  11.00000  0.05    0.13
Q8    1  -0.0793  0.4188  0.1553  11.00000  0.05    0.13
Q9    1   0.5592  0.0600  0.2159  11.00000  0.05    0.13
Q10   1   0.4952  0.4788 -0.1976  11.00000  0.05    0.12
```

```
Q11   1   0.2485  0.3410 -0.0016  11.00000  0.05    0.12
Q12   1   0.2161  0.3580 -0.0402  11.00000  0.05    0.12
Q13   1   0.1025  0.2278  0.0572  11.00000  0.05    0.12
Q14   1   0.4146  0.2413 -0.1243  11.00000  0.05    0.12
Q15   1   0.3293  0.6135  0.0503  11.00000  0.05    0.12
Q16   1   0.0547  0.0127  0.3642  11.00000  0.05    0.11
Q17   1   0.4906  0.8134 -0.0350  11.00000  0.05    0.11
Q18   1   0.3625  0.6778  0.0318  11.00000  0.05    0.11
Q19   1   0.2718  0.2807  0.1701  11.00000  0.05    0.11
Q20   1   0.1723  0.7313  0.1022  11.00000  0.05    0.11
```

Computed structures

stationary points shown in Scheme5 (BP86/SVP+ZPE)

complex of **12** and **1**
E=-1968.7322607

C	-3.6518751	1.6947683	-0.1070387
H	-4.1812431	1.4619721	-1.0554229
C	-3.5161331	0.3906910	0.6964266
H	-4.5446920	0.0227422	0.9154158
N	-2.7471064	0.5803396	1.9290032
N	-2.3847543	2.3576099	-0.4069863
C	-1.4430724	1.8196659	-1.2153158
O	-1.6310541	0.7115637	-1.7716250
C	-0.2130982	2.6492724	-1.5112757
C	0.4873383	3.5153678	-0.6248073
C	0.2585932	2.5413622	-2.8391940
C	1.5954102	4.2480160	-1.0816019
C	1.3604065	3.2786656	-3.2922960
C	2.0279268	4.1414657	-2.4120233
S	0.1768214	3.6762641	1.1773097
O	1.1474843	4.6582138	1.6925154
O	-1.3033291	4.0864304	1.3530927
O	0.2839967	2.2587368	1.7232208
C	-2.4774811	-0.4515556	2.6831309
C	-2.9252093	-1.8232056	2.4249848
C	-1.6450405	-0.2597878	3.9382284
C	-2.3951345	-2.8820619	3.0959029
H	-3.6959842	-2.0003834	1.6613201
C	-0.6203220	-1.3963715	4.0989732
C	-1.2992826	-2.7726062	4.1102230
H	-1.7307483	-3.0111930	5.1131434
H	-0.5528941	-3.5687321	3.9069469
Li	-1.5895640	2.2044484	2.1409051
H	-2.1575369	3.2580192	0.0709727
C	0.1651764	-1.9446808	-0.6352111
C	0.3992808	-1.9558196	-2.0009535

C	0.6330930	-2.9861862	0.2479300
C	1.1637877	-3.0603987	-2.5726317
C	1.4607054	-3.1639478	-3.9515673
C	1.6221062	-4.0724943	-1.6909313
C	2.1978860	-4.2465853	-4.4386795
H	1.0954771	-2.3724226	-4.6209080
C	2.3661297	-5.1637180	-2.1813813
C	2.6499885	-5.2450419	-3.5489888
H	2.4256811	-4.3208318	-5.5123154
H	2.7072606	-5.9289508	-1.4698141
H	3.2318617	-6.0982649	-3.9299136
O	1.3656592	-4.0383659	-0.3545221
O	0.4676606	-3.0662491	1.4555641
O	-0.0246609	-1.0288389	-2.8537199
H	-0.6026265	-0.3253270	-2.3974872
H	-2.7805282	-3.8927861	2.8759933
H	-0.3900205	-1.1229015	-0.1651632
H	-1.1297877	0.7234690	3.9201534
H	-2.3382200	-0.2399562	4.8114343
H	0.0835177	-1.3756288	3.2417875
H	-0.0248289	-1.2440905	5.0211684
H	-3.0303397	-0.3671938	0.0459451
H	-4.2684508	2.4281464	0.4504182
H	-0.2717186	1.8642729	-3.5239336
H	1.6948781	3.1750460	-4.3352500
H	2.8956642	4.7266030	-2.7521832
H	2.1179361	4.8926966	-0.3604832

TS 13

E=-1968.6623721 $\nu_{i=}$161

C	-1.5944016	1.3884092	-1.5026212
H	-1.9315307	1.6187864	-2.5335731
C	-0.6427131	0.1821600	-1.5596471
H	-1.1662824	-0.7052759	-1.9781776
N	-0.0225705	-0.0940837	-0.2349406
N	-0.9772923	2.5934779	-0.9613258
C	-0.6545270	3.6580493	-1.7837995
O	-0.7326367	3.5795199	-3.0098934
C	-0.3022337	4.9732253	-1.1036157
C	0.3477244	5.1984101	0.1425428
C	-0.6986467	6.1081494	-1.8462914
C	0.5438735	6.5067348	0.6181798
C	-0.5100594	7.4112131	-1.3681745
H	-1.1650459	5.9269581	-2.8256665
C	0.1054373	7.6128690	-0.1243221
H	1.0670582	6.6331464	1.5766567
H	-0.8413590	8.2688169	-1.9730286
H	0.2629661	8.6297139	0.2660306
S	1.1329823	3.9087634	1.1800838
O	1.7645637	4.5875160	2.3255178
O	0.0269986	2.8967936	1.5634957

O	2.0695458	3.1632459	0.2328754
C	-0.8335915	-0.6453144	0.7733784
C	-1.8266192	-1.5932763	0.4903781
C	-0.3684181	-0.4328753	2.1829254
C	-2.0720341	-2.5838967	1.4518195
H	-2.1341166	-1.7694040	-0.5491558
C	-1.3400878	-0.9876257	3.2365662
H	0.6509510	-0.8831045	2.2768137
H	-0.2089342	0.6611973	2.3306210
C	-1.8182754	-2.4096151	2.9026679
H	-0.8722140	-0.9532380	4.2391118
H	-2.2218831	-0.3135022	3.2763160
H	-2.7591780	-2.6549872	3.4475293
H	-1.1206328	-3.2267607	3.2310870
Li	0.9653632	1.6435019	0.2124996
H	-1.0031469	2.7690426	0.0621488
C	0.4225138	-3.7128105	0.9990672
C	0.6352833	-3.5651215	-0.4015576
C	0.1536877	-4.9622824	1.6580805
C	0.6016783	-4.8258645	-1.1727785
C	0.8370120	-4.8511372	-2.5661615
C	0.3187137	-6.0431667	-0.5005772
C	0.7953690	-6.0536945	-3.2752121
H	1.0559813	-3.8922747	-3.0583580
C	0.2738003	-7.2574522	-1.2135625
C	0.5112585	-7.2568591	-2.5926340
H	0.9831119	-6.0656168	-4.3590294
H	0.0521614	-8.1831518	-0.6635148
H	0.4759701	-8.2076639	-3.1462806
O	0.0684129	-6.1058994	0.8428743
O	-0.0669925	-5.1133206	2.8561408
O	0.8261501	-2.4543470	-0.9984347
H	0.6372601	-0.9684786	-0.4241313
H	-2.5726006	-3.5104398	1.1254262
H	0.6523440	-2.8754091	1.6673025
H	-2.4879721	1.1374467	-0.8912019
H	0.1921130	0.4027460	-2.2543089

15

E= -1968.7106610

C	0.1239127	1.0259535	-1.6813914
H	0.3687278	1.0531907	-2.7646478
C	1.4137838	0.6493148	-0.9291411
H	1.7928486	-0.3284198	-1.3013953
N	1.2612073	0.6189857	0.5456254
N	-0.4193625	2.3162040	-1.2689120
C	0.1345301	3.4888407	-1.7449435
O	1.1589413	3.4814890	-2.4311256
C	-0.6203895	4.7774276	-1.4694728
C	-1.3833540	5.1316724	-0.3223362
C	-0.5129825	5.7291942	-2.5079458

C	-2.0306759	6.3776323	-0.2574576
C	-1.1676753	6.9660213	-2.4466664
C	-1.9375276	7.2894918	-1.3194383
S	-1.4997977	4.1396673	1.2140051
O	-2.3040810	4.9175184	2.1739875
O	-2.0957182	2.7718650	0.8238555
O	-0.0492979	3.8674115	1.6097859
C	0.8939889	-0.5967648	1.2001138
C	0.3020074	-1.6376849	0.5611702
C	1.1539155	-0.5884494	2.6928706
C	-0.1452392	-2.9035744	1.2525109
H	0.1491943	-1.5871510	-0.5253078
C	1.0448585	-1.9880849	3.3115318
C	-0.1920571	-2.7243457	2.7853206
H	-1.1027331	-2.1337015	3.0264914
H	-0.3037193	-3.6998340	3.2962499
Li	-0.1861154	2.0636466	1.0551503
H	-1.3542967	2.3707511	-0.8203184
C	0.7829343	-4.1337876	0.8065799
C	0.8222009	-4.2122348	-0.7206482
C	0.3004836	-5.4041472	1.4770562
C	-0.2360217	-5.0549525	-1.3243371
C	-0.5158460	-5.0018419	-2.7086262
C	-0.9842359	-5.9337233	-0.5062395
C	-1.5354904	-5.7824612	-3.2586822
H	0.0944190	-4.3225462	-3.3229122
C	-2.0066582	-6.7286875	-1.0524800
C	-2.2812890	-6.6416653	-2.4235549
H	-1.7566501	-5.7303368	-4.3347683
H	-2.5615876	-7.4097241	-0.3917998
H	-3.0852146	-7.2602839	-2.8507642
O	-0.7196119	-6.1025537	0.8355812
O	0.6774782	-5.8111565	2.5497559
O	1.6296194	-3.5626572	-1.3792032
H	-1.1690667	-3.1566543	0.8971837
H	1.8062209	-3.9280614	1.1740883
H	2.1593254	-0.1527080	2.8929518
H	0.4311675	0.1035090	3.1867916
H	1.9591933	-2.5738799	3.0757066
H	1.0072615	-1.9104617	4.4164896
H	2.1692396	1.4218297	-1.1691296
H	-0.6669252	0.2656323	-1.5270841
H	0.1117622	5.4608171	-3.3721805
H	-1.0696332	7.6787573	-3.2793691
H	-2.4552630	8.2584920	-1.2539241
H	-2.5929441	6.6184045	0.6559728
H	2.1006994	1.0062681	0.9957770

TS 14

E=-1968.6794462 $\nu_{i=}$729

C	-2.9015215	1.6763161	-0.9777069
H	-3.1849280	1.5761805	-2.0469826
C	-2.2621869	0.3516098	-0.5256486
H	-3.0172895	-0.4592173	-0.6568757
N	-1.7819026	0.4315297	0.8519827
N	-2.0256882	2.8292712	-0.8099190
C	-0.9912325	3.0636995	-1.6838067
O	-0.7270596	2.2822796	-2.6028929
C	-0.2442287	4.3811508	-1.5337550
C	0.0426301	5.1158885	-0.3490466
C	0.2159871	4.9090176	-2.7607200
C	0.7295038	6.3405581	-0.4235546
C	0.8925950	6.1330760	-2.8338162
C	1.1437613	6.8581477	-1.6595112
S	-0.2826040	4.5474685	1.3672576
O	0.2253052	5.5963852	2.2722365
O	-1.7970650	4.2802399	1.4768482
O	0.3975174	3.1858918	1.4670920
C	-1.3282589	-0.6367914	1.4553457
C	-1.2805255	-1.9707073	0.8514511
C	-0.8515286	-0.4770984	2.8934473
C	-0.8777364	-3.1468819	1.5966057
H	-1.9650736	-2.1428642	0.0042062
C	0.0528924	-1.6170931	3.3785251
C	-0.5653760	-2.9849813	3.0757785
H	-1.5345497	-3.0874413	3.6166105
H	0.0757900	-3.8151619	3.4340704
Li	-1.2894187	2.2836751	1.4424820
H	-2.2629580	3.5692606	-0.1200275
C	0.8791362	-3.7801369	0.8800632
C	0.7890529	-3.4949042	-0.5207966
C	1.1038393	-5.1717032	1.3281755
C	0.7157728	-4.5813780	-1.4745005
C	0.5490787	-4.3778730	-2.8679708
C	0.8273128	-5.9071954	-0.9758950
C	0.5004358	-5.4687041	-3.7360268
H	0.4634584	-3.3447141	-3.2348457
C	0.7823593	-7.0059101	-1.8514282
C	0.6187277	-6.7805387	-3.2235856
H	0.3716467	-5.3093792	-4.8164839
H	0.8728686	-8.0188896	-1.4350589
H	0.5819064	-7.6409434	-3.9087036
O	0.9730046	-6.1754452	0.3582017
O	1.3450527	-5.5193692	2.4640330
O	0.6050076	-2.2595481	-0.8840672
H	-0.1100793	-1.8483880	-0.1161493
H	-1.4322958	-4.0679145	1.3469168
H	1.4343390	-3.0463694	1.4834227
H	-0.3436654	0.5031304	3.0117493

H	-1.7630862	-0.4354884	3.5318826
H	1.0504394	-1.5364751	2.8901564
H	0.2441444	-1.5148075	4.4654527
H	-1.4193231	0.1202857	-1.2157716
H	-3.8232351	1.8706959	-0.3918903
H	0.0216551	4.3134451	-3.6647045
H	1.2265757	6.5170804	-3.8097334
H	1.6751269	7.8213488	-1.6977403
H	0.9430469	6.8667217	0.5177487

16

E=-1968.7221240

C	-2.5488399	1.7441584	-1.2283762
H	-2.7134250	1.7315368	-2.3262258
C	-2.0357639	0.3514368	-0.8188392
H	-2.7781395	-0.4103676	-1.1400404
N	-1.7532087	0.2951166	0.6181102
N	-1.6316916	2.8260572	-0.8923624
C	-0.5581862	3.1108764	-1.7073802
O	-0.2869390	2.4128025	-2.6872495
C	0.2199548	4.3834909	-1.4103238
C	0.4882840	4.9917538	-0.1526349
C	0.7353014	5.0114214	-2.5661719
C	1.2121833	6.1951508	-0.0856367
C	1.4476659	6.2154751	-2.4988879
C	1.6804807	6.8162041	-1.2526680
S	0.0891049	4.2663311	1.4843268
O	0.6134099	5.1935305	2.5049323
O	-1.4383641	4.0573391	1.5272719
O	0.7064224	2.8714413	1.4576591
C	-1.7725473	-0.8077926	1.2923729
C	-2.0469225	-2.1964226	0.7382785
C	-1.4963471	-0.7752161	2.7820328
C	-0.9254987	-3.2024648	1.1239127
H	-2.9899979	-2.5667535	1.1982320
C	-0.4125877	-1.7908712	3.2011462
C	-0.7203267	-3.1910439	2.6552601
H	-1.6611589	-3.5709027	3.1119224
H	0.0736819	-3.9032724	2.9556704
Li	-1.0060903	2.0511750	1.3118325
H	-1.8770777	3.5109168	-0.1502971
C	0.3931774	-2.8989706	0.2975443
C	0.1195757	-3.2034134	-1.1803895
C	1.5750665	-3.6557002	0.8802147
C	0.4736947	-4.5729589	-1.6138313
C	0.0360393	-5.0810234	-2.8585877
C	1.2595776	-5.3961062	-0.7742556
C	0.3515137	-6.3861001	-3.2438082
H	-0.5600415	-4.4122822	-3.4975563
C	1.5871523	-6.7080054	-1.1568014
C	1.1249285	-7.1970676	-2.3859182

H	0.0018345	-6.7798438	-4.2091897
H	2.2093381	-7.3176666	-0.4865012
H	1.3788704	-8.2255952	-2.6841153
O	1.7861440	-4.9507972	0.4192387
O	2.2980445	-3.2390378	1.7516391
O	-0.4262010	-2.3839800	-1.9147547
H	-2.1938373	-2.1738285	-0.3556290
H	-1.2653832	-4.2181329	0.8266903
H	0.6308583	-1.8206537	0.3930373
H	-1.2278281	0.2515404	3.1038038
H	-2.4489691	-1.0314547	3.3018542
H	0.5822718	-1.4587206	2.8397468
H	-0.3423944	-1.8211940	4.3075351
H	-1.1017408	0.1387546	-1.3821761
H	-3.5178418	1.9558921	-0.7316843
H	0.5556069	4.5117288	-3.5292145
H	1.8249827	6.6809388	-3.4221654
H	2.2404733	7.7611468	-1.1816227
H	1.4111411	6.6198452	0.9086909

Enantio-determining transition states shown in Figure3 (TPSS/SVP+ZPE)

14a, pro (R)

E(SCF+ZPE)=-12952.0004768 vi1060.58

C	-0.2181359	-2.8000149	0.0290295
H	0.2816299	-3.4321850	0.7804820
C	-1.7623508	-2.8146626	0.3738354
H	-2.1634050	-3.6721243	-0.1959572
C	0.0443312	-3.3620043	-1.3600645
C	-0.4403323	-2.7192507	-2.5234812
C	0.7695331	-4.5533174	-1.5234748
C	-0.2069672	-3.2488957	-3.7927203
H	-1.0142952	-1.7890783	-2.4365746
C	1.0075038	-5.1043493	-2.7925880
H	1.1631763	-5.0695873	-0.6406039
C	0.5177026	-4.4510331	-3.9393312
H	-0.5769543	-2.7509791	-4.6941140
H	1.5772646	-6.0337752	-2.8723765
C	-2.0571102	-3.0987826	1.8459312
C	-1.7754994	-2.1784635	2.8713651
C	-2.6162350	-4.3432906	2.2087685
C	-2.0430745	-2.4824018	4.2128805
H	-1.3167090	-1.2092168	2.6537767
C	-2.8864299	-4.6619271	3.5408613
H	-2.8358351	-5.0847078	1.4302840

C	-2.6004537	-3.7290146	4.5577058
H	-1.7960537	-1.7415839	4.9772060
H	-3.3162161	-5.6283862	3.8209216
N	-2.3754100	-1.5524874	-0.0706572
N	0.4037979	-1.4517571	0.1427258
O	0.6883505	-4.8918365	-5.2179927
O	-2.8916217	-4.1221520	5.8315711
C	-2.5936233	-3.2300901	6.8999940
H	-2.8954531	-3.7506839	7.8206091
H	-1.5120955	-2.9999203	6.9454469
H	-3.1620796	-2.2847002	6.8102751
C	1.4088940	-6.1003495	-5.4322654
H	1.4155086	-6.2586668	-6.5204853
H	2.4503950	-6.0213873	-5.0668181
H	0.9147334	-6.9592716	-4.9390206
C	1.2856449	-1.1673097	1.1947166
O	1.3237130	-1.8014646	2.2309746
C	2.2849135	-0.0514086	0.8599360
C	1.9583468	1.3231102	0.7978549
C	3.6021903	-0.4634223	0.5666634
C	2.9325755	2.2587455	0.3873866
C	4.6040466	0.4745476	0.2438250
C	4.2640743	1.8416556	0.1476408
S	0.2142373	1.8401228	1.1699494
O	0.2328236	3.1686626	1.7932272
O	-0.4758660	1.7077309	-0.1815305
O	-0.3184052	0.7217726	2.0475681
C	-3.5833655	-1.5241045	-0.5667858
C	-4.0983434	-0.2277699	-1.0263474
C	-4.4620054	-2.7555438	-0.7267468
C	-5.2952771	-0.1129879	-1.8353256
H	-3.3201141	0.5289204	-1.2036173
C	-5.9222856	-2.4347087	-1.0814343
H	-4.0100125	-3.3758275	-1.5277132
H	-4.4132985	-3.3596736	0.1952870
C	-6.0257108	-1.3961023	-2.2028417
H	-6.4467996	-3.3612023	-1.3709500
H	-6.4430858	-2.0577956	-0.1791860
H	-7.0766838	-1.1845540	-2.4629872
H	-5.5438870	-1.7860026	-3.1226010
Li	-1.2461137	0.0116008	0.4346456
H	0.6968337	-1.0539777	-0.7548699
H	-5.2415905	0.6289870	-2.6423656
C	-7.7309426	1.3505942	-1.7037374
C	-6.6892835	0.8629143	-0.7763253
C	-6.8984722	3.6134585	-1.3551250
C	-7.0149093	4.9622908	-1.7205053
C	-5.9593177	3.2077909	-0.3741654
C	-6.1797877	5.9049617	-1.1129023
H	-7.7502267	5.2443367	-2.4788620
C	-5.1206991	4.1795736	0.2237503

C	-5.2294505	5.5180058	-0.1445735
H	-6.2650124	6.9580693	-1.4003174
H	-4.3904922	3.8477378	0.9678870
H	-4.5769280	6.2674576	0.3129595
H	-4.5539873	0.4465311	0.2065577
Br	4.0227556	-2.3216530	0.5573829
Br	6.3774819	-0.0959430	-0.0839199
Br	5.5954263	3.1032069	-0.3219931
Br	2.4898794	4.0741725	0.0687308
H	-6.9907315	-0.0414909	-0.2383662
O	-7.7189460	2.7185418	-1.9960468
O	-5.0162844	1.3331901	0.7868390
C	-5.9034022	1.8026563	-0.0362246
O	-8.5598692	0.6645418	-2.2599611

14b pro (S)

E(SCF+ZPE)=-12951.9963444 νi1250.16

C	1.4221130	-0.8223904	-0.7352191
H	2.4991760	-0.6008724	-0.7972944
C	1.2607276	-2.1461723	0.1151788
H	1.2149619	-2.9709401	-0.6170719
C	0.8678495	-0.9947320	-2.1426009
C	-0.5154221	-1.1617139	-2.3826706
C	1.7299911	-1.0083322	-3.2507926
C	-1.0119214	-1.3363005	-3.6744487
H	-1.2245168	-1.1529756	-1.5457182
C	1.2496890	-1.1874501	-4.5576346
H	2.8062915	-0.8724066	-3.0967841
C	-0.1307683	-1.3542910	-4.7769037
H	-2.0829823	-1.4585128	-3.8619918
H	1.9574320	-1.1877641	-5.3906465
C	2.4343397	-2.4444600	1.0481391
C	3.0637729	-1.4510286	1.8196979
C	2.8846860	-3.7761162	1.1845769
C	4.1097510	-1.7673241	2.6996506
H	2.7732439	-0.3984438	1.7400404
C	3.9255464	-4.1042647	2.0527462
H	2.4138409	-4.5748527	0.6005185
C	4.5480196	-3.0992265	2.8225594
H	4.5715203	-0.9607434	3.2744895
H	4.2783502	-5.1349868	2.1533794
N	0.0324818	-2.0592527	0.9292508
N	0.8183739	0.3639932	-0.0800163
O	-0.7080257	-1.5348492	-5.9980875
O	5.5566288	-3.5108480	3.6425198
C	6.2153693	-2.5373835	4.4464515
H	6.9775637	-3.0854102	5.0191661
H	6.7065440	-1.7648215	3.8251370
H	5.5115571	-2.0465637	5.1452835
C	0.1258005	-1.5442039	-7.1516558
H	-0.5465931	-1.6939776	-8.0087281

H	0.6635324	-0.5844094	-7.2699609
H	0.8613093	-2.3707811	-7.1130999
C	1.5936102	1.4893826	0.2146467
O	2.8088867	1.4681296	0.2916380
C	0.7683520	2.7765650	0.3240288
C	-0.0708695	3.0922437	1.4167397
C	0.8396162	3.6607996	-0.7732370
C	-0.8771228	4.2503698	1.3715189
C	0.1063262	4.8649922	-0.7855447
C	-0.7627767	5.1554342	0.2889855
S	-0.1464682	1.9194228	2.8543943
O	-0.3348665	2.6972657	4.0842210
O	-1.2562325	0.9563097	2.4510865
O	1.1573541	1.1466269	2.7699131
C	-0.9326745	-2.9262675	0.8643911
C	-1.0063341	-4.0983700	-0.0418617
C	-2.1189462	-2.7475839	1.7944039
C	-2.2610022	-4.7948728	-0.2007017
H	-0.3871596	-4.0608898	-0.9495984
C	-3.4327098	-2.8642025	1.0003558
H	-2.0832304	-3.5287659	2.5815197
H	-2.0495765	-1.7707004	2.3005779
C	-3.5780863	-4.2432670	0.3292370
H	-4.2943810	-2.6778339	1.6624738
H	-3.4480673	-2.0684458	0.2319451
H	-4.2679306	-4.1789492	-0.5326443
H	-4.0604296	-4.9652435	1.0147832
Li	0.1419903	-0.3714246	2.0109613
H	-0.1420559	0.5776758	-0.3614498
C	-2.0365560	-6.5763955	1.0389627
C	-0.7489062	-6.9649758	0.5804212
C	-3.2102145	-7.4218182	0.8182546
C	-0.6394093	-8.1350171	-0.2796941
C	0.5864932	-8.5671107	-0.8359447
C	-1.8177290	-8.8725194	-0.5512045
C	0.6327100	-9.7055721	-1.6376227
H	1.4858293	-7.9849290	-0.6145122
C	-1.7734318	-10.0219232	-1.3563075
C	-0.5498961	-10.4304713	-1.8936013
H	1.5825222	-10.0388135	-2.0667957
H	-2.6999161	-10.5704032	-1.5462287
H	-0.5139534	-11.3267778	-2.5217077
O	-3.0431217	-8.5029129	-0.0605558
O	-4.3150453	-7.2431076	1.2865980
O	0.2544230	-6.1800499	0.7977934
H	-0.2256070	-5.0581977	0.5544551
H	-2.3470277	-5.3858417	-1.1198391
H	-2.0967435	-5.9459804	1.9287982
Br	-2.1834121	4.5950642	2.7003979
Br	1.9044160	3.1958518	-2.2821241
Br	0.2638533	6.0640301	-2.2399481

Br -1.7825399 6.7498746 0.2604944

14c, pro(S)
E(SCF+ZPE)=-12951.9947666 vi1175.18

C	-0.6877103	-2.8147263	-0.3244040
H	-0.0130785	-3.6919023	-0.2868339
C	-1.4983319	-2.8071212	1.0360446
H	-2.1350346	-3.7099174	0.9961590
C	-1.6419597	-2.9989751	-1.4941579
C	-2.2308561	-1.9369301	-2.2117753
C	-2.0411465	-4.3082474	-1.8099387
C	-3.1954324	-2.1815479	-3.1898847
H	-1.9038284	-0.9039023	-2.0574754
C	-3.0075442	-4.5723669	-2.7925632
H	-1.5836309	-5.1566411	-1.2854043
C	-3.5984159	-3.4995565	-3.4865676
H	-3.6408643	-1.3566005	-3.7531113
H	-3.2779898	-5.6085380	-3.0120245
C	-0.5786425	-2.9269856	2.2521335
C	-0.1673905	-1.8142781	3.0078886
C	-0.0882959	-4.1985460	2.6264645
C	0.7077511	-1.9528920	4.0984285
H	-0.5392269	-0.8150244	2.7571391
C	0.7771480	-4.3528789	3.7073488
H	-0.3978300	-5.0868387	2.0626314
C	1.1864643	-3.2270985	4.4553819
H	1.0005807	-1.0614963	4.6591007
H	1.1505911	-5.3381318	4.0019345
N	-2.2761208	-1.5711928	1.0663396
N	0.1213186	-1.5779788	-0.3370498
O	-4.5477590	-3.6344191	-4.4592123
O	2.0301708	-3.4781370	5.4946996
C	2.4807911	-2.3840888	6.2873920
H	3.1450959	-2.8177464	7.0488611
H	3.0452239	-1.6510338	5.6808090
H	1.6362927	-1.8710541	6.7855259
C	-4.9593875	-4.9437241	-4.8270988
H	-5.7074079	-4.8144671	-5.6231672
H	-4.1124856	-5.5426302	-5.2144557
H	-5.4214873	-5.4805776	-3.9753381
C	0.8601832	-1.1111880	-1.4317197
O	0.7225986	-1.4799972	-2.5791037
C	1.9554953	-0.1098997	-1.0172661
C	1.7288953	1.2461637	-0.6822583
C	3.2700782	-0.6185672	-0.9824451
C	2.8112912	2.0589910	-0.2796072
C	4.3664010	0.2063188	-0.6608427
C	4.1333181	1.5532641	-0.3082368
S	-0.0171664	1.8770996	-0.6345033
O	-0.0394579	3.2932840	-1.0278850
O	-0.7808866	0.9502517	-1.5585515

O	-0.4447359	1.5420283	0.7892370
C	-3.5103294	-1.5101194	1.4673789
C	-4.1269793	-0.1653822	1.4687140
C	-4.3579061	-2.6908277	1.9231106
C	-5.5057748	0.0595779	1.8062279
H	-3.4232922	0.6397334	1.7295808
C	-5.5873833	-2.2566810	2.7410036
H	-4.6839064	-3.2479889	1.0213833
H	-3.7272971	-3.3829113	2.5072800
C	-6.3856236	-1.1685535	1.9938896
H	-6.2362247	-3.1293910	2.9304664
H	-5.2643912	-1.8756009	3.7274979
H	-7.3126137	-0.8967813	2.5230764
H	-6.6907255	-1.5839701	1.0155080
Li	-1.4131120	-0.0185888	0.0827153
H	0.5941829	-1.4210655	0.5614246
C	-6.3875650	1.0429780	0.1619095
C	-5.2434012	1.6239061	-0.4597917
C	-7.4759161	1.8964524	0.6715749
C	-5.0568525	3.0593335	-0.4171296
C	-3.8828827	3.6957681	-0.8882799
C	-6.0989496	3.8411648	0.1400450
C	-3.7669395	5.0819056	-0.8162996
H	-3.0728466	3.0801393	-1.2905237
C	-5.9884876	5.2378608	0.1986846
C	-4.8239167	5.8473541	-0.2811429
H	-2.8513643	5.5674865	-1.1662028
H	-6.8088430	5.8179801	0.6298200
H	-4.7328861	6.9374311	-0.2284588
O	-7.2387107	3.2775012	0.6624262
O	-8.5276401	1.5064720	1.1271113
O	-4.2985601	0.8280463	-0.8581598
H	-4.1611538	0.1212107	0.0968635
H	-5.7164776	0.9069792	2.4679968
H	-6.7257259	0.0787624	-0.2234895
Br	3.5584182	-2.4719331	-1.3256863
Br	6.1246579	-0.4904545	-0.6776423
Br	5.5972463	2.6641179	0.1474126
Br	2.5406488	3.8167512	0.3790070

14d, pro(R)

E(SCF+ZPE)=-12952.0003504 vi1215.96

C	0.9091555	-0.9583193	0.5159365
H	1.2583565	-0.6693148	1.5204012
C	-0.1413171	-2.1218841	0.7291033
H	0.4516679	-3.0524697	0.6554316
C	2.1034993	-1.4039743	-0.3140897
C	3.4073935	-1.3267074	0.2199200
C	1.9571956	-1.8970997	-1.6239946
C	4.5194566	-1.7285515	-0.5199793

H	3.5507005	-0.9418762	1.2358220
C	3.0647642	-2.3002011	-2.3848699
H	0.9584125	-1.9784176	-2.0681343
C	4.3580191	-2.2194017	-1.8320753
H	5.5305085	-1.6688865	-0.1061921
H	2.9077303	-2.6777895	-3.3985196
C	-0.7593736	-2.1076857	2.1267391
C	-1.7545811	-1.1916610	2.5100613
C	-0.2838032	-3.0170644	3.0964195
C	-2.2628439	-1.1791651	3.8162426
H	-2.1372437	-0.4485208	1.8039079
C	-0.7777286	-3.0141275	4.4022756
H	0.4982349	-3.7376921	2.8251824
C	-1.7734049	-2.0884039	4.7740556
H	-3.0258955	-0.4412292	4.0753312
H	-0.4060236	-3.7154737	5.1552952
N	-1.1620387	-2.0909208	-0.3346448
N	0.3234251	0.2747290	-0.0844787
O	5.5012389	-2.5869425	-2.4773860
O	-2.1931468	-2.1488105	6.0710508
C	-3.1636684	-1.2059873	6.5132764
H	-3.3339807	-1.4252097	7.5775449
H	-2.7972809	-0.1674972	6.4054218
H	-4.1159395	-1.3133331	5.9595136
C	5.4048656	-3.0844250	-3.8071520
H	6.4348712	-3.3085435	-4.1207804
H	4.7984376	-4.0092676	-3.8508277
H	4.9689961	-2.3313173	-4.4912144
C	0.0975997	1.4011814	0.7246352
O	0.0197311	1.3525925	1.9359366
C	0.0818984	2.7168980	-0.0638123
C	-0.9943419	3.1482086	-0.8724069
C	1.2454914	3.5104468	0.0209348
C	-0.8727123	4.3329889	-1.6304593
C	1.3407174	4.7394379	-0.6634274
C	0.2777061	5.1476385	-1.4991796
S	-2.5047101	2.0761500	-0.9922065
O	-3.6868066	2.9345824	-1.1262497
O	-2.1635298	1.1491904	-2.1514351
O	-2.4578338	1.2335430	0.2700745
C	-1.7754317	-3.1784246	-0.6999946
C	-1.5424582	-4.5122261	-0.1007080
C	-2.8422928	-3.0558011	-1.7774530
C	-2.3352496	-5.6506302	-0.4942180
C	-3.0930350	-4.3683570	-2.5337680
C	-3.4042466	-5.5071341	-1.5557033
Li	-1.6991694	-0.2314805	-0.8261345
H	0.7134543	0.5062682	-1.0036972
C	-0.9994937	-6.9771948	-1.5193863
C	0.1775473	-6.7817501	-0.7456791
C	-1.6554264	-8.2886838	-1.5828106

C	0.6405921	-7.8579287	0.1159301
C	1.7641985	-7.7331785	0.9658519
C	-0.0693204	-9.0831657	0.0854911
C	2.1679512	-8.8057301	1.7582953
H	2.2983506	-6.7783948	0.9755075
C	0.3370535	-10.1661150	0.8808256
C	1.4517929	-10.0203080	1.7113557
H	3.0373262	-8.7083642	2.4154874
H	-0.2319300	-11.0985575	0.8355710
H	1.7690439	-10.8636299	2.3334726
O	-1.1792594	-9.2673256	-0.6983705
O	-2.5922280	-8.5840038	-2.2935884
O	0.7043360	-5.5992968	-0.7028779
H	-0.2671088	-4.8827316	-0.5457711
H	-4.3460465	-5.2821198	-1.0119656
H	-3.5740412	-6.4664684	-2.0753717
H	-2.5487737	-6.3846698	0.2914289
H	-1.1186871	-6.3699911	-2.4200052
H	-1.2511435	-4.4906272	0.9591183
H	-3.7797034	-2.7513735	-1.2682783
H	-2.5805052	-2.2351192	-2.4674082
H	-2.2043924	-4.6265743	-3.1429443
H	-3.9272594	-4.2401421	-3.2434141
Br	2.7306812	2.8855133	1.0385867
Br	2.8858290	5.8154083	-0.4841594
Br	0.4128936	6.7770963	-2.4530869
Br	-2.1926215	4.8334333	-2.8948707

14e, pro(R)

E(SCF+ZPE)=-12951.9776499 vi1221.36

C	-0.2304951	-1.8226838	-0.1677179
H	-0.0317479	-0.7568385	-0.3700137
C	-1.3595567	-2.1983400	-1.2122325
H	-0.7899100	-2.1292231	-2.1552778
C	-0.5717809	-1.9146000	1.3194980
C	-0.0580912	-2.9320244	2.1511143
C	-1.3810780	-0.9310061	1.9157479
C	-0.3821677	-2.9946044	3.5068750
H	0.6256808	-3.6740570	1.7272249
C	-1.7202517	-0.9786731	3.2775165
H	-1.7449725	-0.0858686	1.3213152
C	-1.2279705	-2.0235462	4.0826482
H	0.0235159	-3.7800746	4.1514985
H	-2.3436013	-0.1851851	3.6982497
C	-1.9406701	-3.6131407	-1.2266639
C	-2.0357677	-4.4845998	-0.1282445
C	-2.4141187	-4.0899023	-2.4731689
C	-2.5885618	-5.7698606	-0.2494474
H	-1.6864548	-4.1663354	0.8564376
C	-2.9822081	-5.3549926	-2.6105558

H	-2.3319695	-3.4482952	-3.3586307
C	-3.0759330	-6.2123270	-1.4932665
H	-2.6338918	-6.4104557	0.6352110
H	-3.3463086	-5.7142995	-3.5776361
N	-2.2950290	-1.0484966	-1.3236975
N	0.9846287	-2.5349736	-0.5608050
O	-1.4906927	-2.1682578	5.4133940
O	-3.6400754	-7.4310988	-1.7189015
C	-3.7442372	-8.3450503	-0.6320847
H	-4.2187794	-9.2482638	-1.0419309
H	-2.7484381	-8.6050990	-0.2257459
H	-4.3741825	-7.9360963	0.1813370
C	-2.3108598	-1.1978642	6.0533345
H	-2.3850199	-1.5103169	7.1051389
H	-1.8604465	-0.1882147	6.0016513
H	-3.3243746	-1.1661677	5.6085413
C	2.1342775	-1.9298691	-1.0327784
O	2.9485375	-2.5036124	-1.7369136
C	2.4870491	-0.5565008	-0.4313452
C	2.2716504	0.6881434	-1.0612827
C	3.2349586	-0.5843410	0.7691917
C	2.7992031	1.8755737	-0.5064833
C	3.7297380	0.5979098	1.3552999
C	3.5154791	1.8360673	0.7090476
S	1.2805878	0.8097992	-2.6076933
O	2.1783345	1.2757864	-3.6705902
O	0.6613084	-0.5709443	-2.7959686
O	0.1091700	1.7185370	-2.2393172
C	-3.5007664	-0.9643140	-0.8473444
C	-4.2214437	0.3096288	-1.1277501
C	-4.2407459	-2.0350370	-0.0609958
C	-5.6320338	0.4513781	-0.8802723
H	-3.8432812	0.8432446	-2.0133954
C	-5.4993494	-1.5271359	0.6616537
H	-4.5172265	-2.8294079	-0.7809408
H	-3.5544525	-2.5041015	0.6592084
C	-6.3978658	-0.6903312	-0.2539397
H	-6.0610558	-2.3879166	1.0616900
H	-5.2001772	-0.9219263	1.5396230
H	-7.2940909	-0.3174712	0.2720172
H	-6.7693858	-1.3160317	-1.0935407
Li	-1.1105733	0.2545150	-2.3195038
H	0.8633292	-3.4777602	-0.9404417
H	-6.1942188	1.0879999	-1.5729027
C	-7.1946275	2.3829313	0.6937762
C	-5.8124804	1.8949861	0.7295266
C	-6.3453860	4.4975695	-0.1635800
C	-6.6749692	5.7851883	-0.6137418
C	-4.9939045	4.0807217	-0.0924868
C	-5.6497364	6.6534933	-0.9997105
H	-7.7275835	6.0776921	-0.6571245

C	-3.9738874	4.9741529	-0.4935722
C	-4.2987152	6.2520695	-0.9441513
H	-5.9049771	7.6583087	-1.3523428
H	-2.9364464	4.6307694	-0.4370144
H	-3.5091828	6.9428284	-1.2547852
H	-3.6872460	1.1914789	-0.2323801
H	-5.6357397	1.0795006	1.4338175
O	-7.3865758	3.6726595	0.1824746
O	-3.5236477	2.2457643	0.4084613
C	-4.7180598	2.7425005	0.4086258
O	-8.1794460	1.7574490	1.0273896
Br	3.6197461	-2.2570809	1.5930610
Br	4.6866275	0.5325151	2.9864550
Br	4.1937413	3.4289570	1.4754118
Br	2.5689131	3.5397951	-1.3824196

14f, pro (S)

E(SCF+ZPE)=-12951.9742793 νi1191.21

C	1.0789169	2.0087392	-0.8155619
H	0.1754162	2.5318403	-0.4760411
C	1.7299645	1.3130762	0.4138420
H	2.5744789	0.6877824	0.0804313
C	1.9914415	3.0498479	-1.4561171
C	3.3747642	2.8372086	-1.6405042
C	1.4309742	4.2417996	-1.9494957
C	4.1703479	3.7859973	-2.2845741
H	3.8502644	1.9209939	-1.2729677
C	2.2178818	5.2041191	-2.5973457
H	0.3534007	4.4047214	-1.8387875
C	3.5978585	4.9807066	-2.7679716
H	5.2439928	3.6282997	-2.4244552
H	1.7434689	6.1169865	-2.9669192
C	2.2510455	2.3171261	1.4461116
C	1.5318281	3.4809259	1.7811094
C	3.4332493	2.0405269	2.1657870
C	1.9679259	4.3459972	2.7985917
H	0.6245244	3.7613069	1.2286797
C	3.8833136	2.8948239	3.1718475
H	4.0111441	1.1391332	1.9323979
C	3.1531587	4.0565987	3.5010405
H	1.3845351	5.2445709	3.0152687
H	4.8043979	2.6856566	3.7242865
N	0.7217891	0.5069506	1.1459365
N	0.6433105	1.0587675	-1.8832437
O	4.4544751	5.8483433	-3.3810261
O	3.6713858	4.8268285	4.4978311
C	2.9894491	6.0238916	4.8584110
H	3.5845647	6.4790079	5.6632420
H	2.9264881	6.7255695	4.0052086
H	1.9690335	5.8119493	5.2308157

C	3.9299081	7.0637228	-3.9027890
H	4.7833291	7.5934669	-4.3509905
H	3.1652276	6.8749193	-4.6801877
H	3.4867654	7.6900698	-3.1049175
C	-0.6668646	0.8175321	-2.3143210
O	-0.9505565	0.7927388	-3.5012969
C	-1.6742341	0.1951545	-1.3313915
C	-2.5736128	0.8933271	-0.4980228
C	-1.7784217	-1.2170997	-1.3981962
C	-3.5044030	0.1919773	0.3008663
C	-2.7541410	-1.9189387	-0.6625122
C	-3.6211131	-1.2108302	0.2011216
S	-2.4693128	2.7420169	-0.4056253
O	-3.8325995	3.2908201	-0.4217495
O	-1.7506779	2.9217031	0.9435747
O	-1.5907164	3.1081833	-1.5528747
C	0.7968239	-0.7859668	1.2273696
C	1.7760433	-1.6211814	0.4722236
C	-0.1951069	-1.5058966	2.1237866
C	1.7703890	-3.0488235	0.6185299
H	1.9593606	-1.2657118	-0.5524064
C	0.3667618	-2.8147495	2.7025052
C	0.8426599	-3.7430456	1.5793639
H	-0.0274102	-4.0647340	0.9669511
H	1.2967030	-4.6715952	1.9697911
Li	-0.5964305	1.8626900	1.8396551
H	1.2037905	1.2034776	-2.7298896
C	3.7235215	-3.5016015	1.6811855
C	4.5241916	-2.5571145	0.9969510
C	3.9362629	-4.9403933	1.5447866
C	5.5163760	-3.0466556	0.0472984
C	6.3335295	-2.1862730	-0.7202962
C	5.6677009	-4.4474654	-0.0933038
C	7.2794669	-2.7115400	-1.5984198
H	6.2001579	-1.1070313	-0.5992202
C	6.6222333	-4.9799441	-0.9746218
C	7.4211433	-4.1094851	-1.7204345
H	7.9115505	-2.0437948	-2.1918368
H	6.7144411	-6.0659693	-1.0603813
H	8.1649770	-4.5233669	-2.4092285
O	4.8924150	-5.3409539	0.5987568
O	3.3375771	-5.8121594	2.1410077
O	4.2522226	-1.2952500	1.1093196
H	2.9965353	-1.2878264	0.9359126
H	2.0858438	-3.6345602	-0.2521311
H	3.2158518	-3.1917414	2.5967351
H	1.2049979	-2.5926469	3.3913144
H	-0.4068517	-3.3197643	3.3045605
H	-0.5271226	-0.8233703	2.9252346
H	-1.0872154	-1.7419123	1.5121897
Br	-0.5824813	-2.2015520	-2.5114717

Br	-2.8925962	-3.8057300	-0.7978347
Br	-4.9032199	-2.1499695	1.2323789
Br	-4.5754780	1.1053069	1.5763924

14g, pro(S)

E(SCF+ZPE)=-12951.9968688 νi1208.20

C	1.3458915	-0.5279370	0.1610886
H	0.9139564	0.3995276	-0.2486123
C	2.0940452	-0.1522662	1.4891527
H	2.7352347	-1.0176321	1.7549287
C	2.3255552	-1.1022014	-0.8533644
C	2.9335649	-2.3618573	-0.6627163
C	2.6772200	-0.3680875	-1.9955730
C	3.8570269	-2.8652085	-1.5771634
H	2.6717388	-2.9748432	0.2086439
C	3.6013498	-0.8584669	-2.9302013
H	2.2218138	0.6138721	-2.1666352
C	4.1991282	-2.1153522	-2.7229314
H	4.3252747	-3.8438921	-1.4359464
H	3.8409037	-0.2559307	-3.8099102
C	3.0029786	1.0543189	1.2663438
C	2.4537643	2.3308486	1.0415176
C	4.4064029	0.9211181	1.2692971
C	3.2771798	3.4475563	0.8370845
H	1.3654097	2.4628839	1.0132318
C	5.2382844	2.0232543	1.0640368
H	4.8576238	-0.0656879	1.4266526
C	4.6789058	3.2997451	0.8502861
H	2.8141735	4.4224431	0.6635687
H	6.3277453	1.9225360	1.0623999
N	1.1121444	0.0930749	2.5632992
N	0.2019084	-1.4112254	0.4915640
O	5.1069239	-2.6902212	-3.5622083
O	5.5676646	4.3180702	0.6661412
C	5.0630295	5.6295440	0.4396244
H	5.9449878	6.2761858	0.3230754
H	4.4498355	5.6749258	-0.4803965
H	4.4586893	5.9851453	1.2960805
C	5.4702162	-1.9933698	-4.7487950
H	6.1940734	-2.6382714	-5.2680527
H	4.5933388	-1.8239348	-5.4022077
H	5.9454895	-1.0205332	-4.5180719
C	-0.8642239	-1.7679559	-0.3472766
O	-1.5461970	-2.7502551	-0.1244039
C	-1.0678007	-0.8946622	-1.5896965
C	-1.6155301	0.4083143	-1.5622413
C	-0.6837676	-1.4554200	-2.8278151
C	-1.6553505	1.1817970	-2.7427793
C	-0.8077770	-0.7215789	-4.0258849
C	-1.2750002	0.6102976	-3.9793999

S	-2.2249807	1.1026600	0.0447326
O	-2.4544549	-0.1123773	0.9240366
O	-0.9996904	1.8206549	0.6041552
O	-3.4175818	1.9119342	-0.2318019
C	1.4743138	0.0845066	3.8136663
C	0.4370561	0.4011528	4.8221470
C	2.8879152	-0.1851604	4.2961725
C	0.7736219	0.6485450	6.2006629
H	-0.4023833	0.9905457	4.4229748
C	2.9921347	-0.4785656	5.8010586
C	2.2202865	0.5479564	6.6373778
H	2.6612845	1.5572919	6.4958691
H	2.2879825	0.3287709	7.7171939
Li	-0.7393049	0.2409209	1.8004132
H	0.3903007	-2.1669036	1.1575220
C	-0.0549136	-0.9499297	7.3265121
C	-1.2512745	-1.1811446	6.5908951
C	-0.1025653	-0.5189428	8.7304041
C	-2.5268551	-0.8251418	7.1874516
C	-3.7573085	-0.9485779	6.5004338
C	-2.5226193	-0.3418279	8.5192815
C	-4.9500145	-0.6057354	7.1334234
H	-3.7390602	-1.3168448	5.4703375
C	-3.7233872	-0.0014345	9.1604316
C	-4.9279608	-0.1361773	8.4636034
H	-5.9012355	-0.6993101	6.6010846
H	-3.6889983	0.3673279	10.1890176
H	-5.8660242	0.1310112	8.9612106
O	-1.3626043	-0.1704000	9.2330433
O	0.8509912	-0.3995630	9.4702794
O	-1.1535017	-1.5356741	5.3486883
H	-0.3040835	-0.7849281	4.9311612
H	0.1751207	1.4067427	6.7187465
H	0.8132650	-1.5653654	7.0781166
H	3.3358829	-1.0061126	3.7079398
H	3.4829167	0.7143015	4.0399663
H	4.0529508	-0.4926410	6.1024466
H	2.6011325	-1.4937090	6.0087492
Br	-2.1282915	3.0175615	-2.7002417
Br	-1.3835860	1.6223353	-5.5767881
Br	-0.3225929	-1.5038190	-5.6794989
Br	0.0245628	-3.2183196	-2.8831091

14h, pro(R)

E(SCF+ZPE)=-12951.9681151 vi1032.41

C	1.5470895	1.9543931	-0.7089262
H	0.7910042	2.6597784	-0.3347313
C	1.9145315	0.9765515	0.4505611
H	2.5600632	0.1784915	0.0432105
C	2.7374771	2.7810791	-1.1840881

C	4.0058031	2.2086745	-1.4229585
C	2.5631342	4.1468539	-1.4667811
C	5.0644959	2.9764582	-1.9098580
H	4.1802500	1.1447973	-1.2256668
C	3.6177701	4.9320043	-1.9544618
H	1.5736800	4.5943413	-1.3240859
C	4.8798996	4.3486778	-2.1765637
H	6.0504344	2.5379281	-2.0914082
H	3.4401036	5.9905428	-2.1618132
C	2.6532147	1.6950135	1.5801579
C	2.1475614	2.8845729	2.1401301
C	3.8407602	1.1661820	2.1267697
C	2.7893846	3.5226885	3.2125359
H	1.2519458	3.3646271	1.7222866
C	4.4942084	1.7889587	3.1916638
H	4.2775184	0.2561484	1.6986030
C	3.9706678	2.9738080	3.7488820
H	2.3672703	4.4507392	3.6061909
H	5.4209556	1.3826950	3.6075095
N	0.6678157	0.4120192	1.0206430
N	0.9625086	1.2799498	-1.8995596
O	5.9737878	5.0177926	-2.6457218
O	4.6757699	3.5108587	4.7854159
C	4.2066721	4.7199213	5.3718799
H	4.9197552	4.9631064	6.1729511
H	4.1855995	5.5457690	4.6354033
H	3.1960839	4.5947588	5.8059918
C	5.8441834	6.4019831	-2.9464429
H	6.8318839	6.7272384	-3.3049997
H	5.0904051	6.5768500	-3.7379133
H	5.5689873	6.9888622	-2.0488281
C	-0.3430364	1.3269982	-2.3648421
O	-0.6029528	1.3821693	-3.5585108
C	-1.4988511	0.9447557	-1.4230425
C	-2.3004162	1.8419954	-0.6829573
C	-1.8794768	-0.4190977	-1.4687896
C	-3.4273100	1.3715794	0.0309794
C	-3.0360574	-0.8833780	-0.8144198
C	-3.8203143	0.0179124	-0.0595455
S	-1.7323123	3.6071130	-0.4981907
O	-2.8942210	4.5041020	-0.5605707
O	-1.1021240	3.5007084	0.9069735
O	-0.6977975	3.7733754	-1.5551143
C	0.5862749	-0.8440589	1.3734292
C	1.6655695	-1.8324089	1.3134466
C	-0.7268975	-1.3335297	1.9574319
C	1.5072410	-3.1533051	1.9065202
C	-0.4669687	-2.1850629	3.2098108
C	0.3165855	-3.4525212	2.8368758
Li	-0.6154900	1.8561631	1.5108536
H	1.5846604	1.3498355	-2.7106275

C	1.2465581	-4.4365077	0.4241709
C	2.3191566	-4.0609917	-0.4497340
C	1.2054397	-5.8109128	0.9661826
C	3.4830347	-4.9121894	-0.5638886
C	4.6266776	-4.5621750	-1.3227379
C	3.4634785	-6.1526875	0.1233734
C	5.7148076	-5.4279660	-1.3908786
H	4.6247820	-3.6017060	-1.8464579
C	4.5587519	-7.0270332	0.0528460
C	5.6761268	-6.6592208	-0.7019216
H	6.5978795	-5.1567257	-1.9769724
H	4.5130073	-7.9761414	0.5934569
H	6.5318106	-7.3400962	-0.7570379
O	2.3943953	-6.5515862	0.8817208
O	0.2601010	-6.3292198	1.5139571
O	2.2951421	-2.8691324	-0.9629630
H	1.8472112	-2.2163849	-0.1349449
H	0.1001791	-1.5852976	3.9462005
H	-1.4209142	-2.4692302	3.6860836
H	0.7069438	-3.9471676	3.7425619
H	-0.3697311	-4.1860955	2.3784802
H	-1.3809167	-0.4723293	2.1818542
H	-1.2621487	-1.9417050	1.2023017
H	2.4538188	-3.5744909	2.2669392
H	0.2537665	-4.0613852	0.1550603
H	2.6859868	-1.4320955	1.3408191
Br	-4.4115737	2.5261920	1.1758587
Br	-5.3645302	-0.6036839	0.8454241
Br	-3.5240426	-2.7140242	-0.9061595
Br	-0.7874341	-1.6613898	-2.4130998

Transition structures **14a-h** (BP86/SVP+ZPE)

TS 14a pro (R)
E(SCF+ZPE)=-12953.2812955 $\nu_{i=}$810

C	-0.7665027	-2.6999797	-0.5102419
H	-0.1621892	-3.4106268	0.0879811
C	-2.1476099	-2.5328458	0.2496947
H	-2.8107987	-3.2946110	-0.2135299
C	-0.9628845	-3.2679984	-1.9078578
C	-1.7345729	-2.5882183	-2.8823173
C	-0.3806733	-4.4957955	-2.2712081
C	-1.9124309	-3.1166095	-4.1633286
H	-2.2123987	-1.6276074	-2.6303024
C	-0.5539930	-5.0455401	-3.5533454
H	0.2278840	-5.0441191	-1.5348836
C	-1.3240312	-4.3545980	-4.5123682

H	-2.5096126	-2.5902475	-4.9222696
H	-0.0816489	-6.0078107	-3.7927017
C	-2.0731409	-2.8843087	1.7367134
C	-1.3818607	-2.0911194	2.6745072
C	-2.7094040	-4.0574519	2.2055408
C	-1.3340230	-2.4449362	4.0323993
H	-0.8411875	-1.1832427	2.3668422
C	-2.6685525	-4.4246413	3.5543714
H	-3.2441277	-4.7077710	1.4930880
C	-1.9802378	-3.6152884	4.4849154
H	-0.7753725	-1.8005478	4.7240902
H	-3.1628712	-5.3380485	3.9160833
N	-2.6750588	-1.1788994	0.0365931
N	0.0371187	-1.4524244	-0.5876336
O	-1.5577713	-4.7905989	-5.7810828
O	-1.9934979	-4.0434929	5.7788548
C	-1.3048706	-3.2761527	6.7538086
H	-1.4444744	-3.8005301	7.7171226
H	-0.2162358	-3.2069302	6.5343943
H	-1.7183692	-2.2464466	6.8396148
C	-0.9895477	-6.0242943	-6.1920376
H	-1.3019883	-6.1760798	-7.2414108
H	0.1221397	-6.0044398	-6.1456843
H	-1.3596935	-6.8761150	-5.5786317
C	1.2160937	-1.3197959	0.1594674
O	1.4869296	-2.0139119	1.1218823
C	2.1951620	-0.2842708	-0.4179358
C	2.0545558	1.1182174	-0.2760439
C	3.2980012	-0.8010112	-1.1360591
C	2.9791671	1.9863231	-0.9026310
C	4.2754019	0.0560018	-1.6898065
C	4.1105718	1.4571643	-1.5762514
S	0.5788972	1.7802307	0.6483865
O	0.9631742	3.0147983	1.3495470
O	-0.4821321	1.8912398	-0.4430657
O	0.1624496	0.6297156	1.5518263
C	-3.9602321	-0.9427887	-0.0337958
C	-4.3821432	0.4439975	-0.2730115
C	-5.0300815	-2.0155771	0.0868965
C	-5.7459104	0.7986096	-0.6017745
H	-3.5916252	1.0957998	-0.6886403
C	-6.4538397	-1.4641063	0.2582028
H	-4.9718613	-2.6367441	-0.8368647
H	-4.7745587	-2.6957124	0.9244247
C	-6.7593427	-0.3279040	-0.7212331
H	-7.1911022	-2.2833901	0.1394845
H	-6.5819405	-1.0982108	1.3020831
H	-7.7905560	0.0586441	-0.5911019
H	-6.6992722	-0.7034682	-1.7690099
Li	-1.2560210	0.2037841	0.2294473
H	0.0906598	-1.0406748	-1.5268025

H	-5.8544058	1.5957662	-1.3566342
C	-7.6994503	2.6039240	0.4287742
C	-6.5036801	1.8839634	0.9137710
C	-6.4425901	4.6837959	0.5848808
C	-6.4545393	6.0714051	0.3636822
C	-5.3008569	4.0516459	1.1465626
C	-5.3202260	6.8250245	0.6910057
H	-7.3535145	6.5314842	-0.0700313
C	-4.1613521	4.8337696	1.4638877
C	-4.1709164	6.2102108	1.2366718
H	-5.3276528	7.9111376	0.5136747
H	-3.2798164	4.3258630	1.8815344
H	-3.2844702	6.8142178	1.4782036
H	-4.2628229	1.0760259	1.0688949
Br	3.4436655	-2.6848541	-1.3963879
Br	5.7769785	-0.6626457	-2.5933826
Br	5.3994682	2.6151597	-2.3447813
Br	2.7122571	3.8651902	-0.9292056
H	-6.7562014	0.9823018	1.4917186
O	-7.5642342	3.9826131	0.2256365
O	-4.3357595	1.9520828	1.7967727
C	-5.3632650	2.6218967	1.3672161
O	-8.7667831	2.0966663	0.1543905

TS 14b pro (S)

E(SCF+ZPE)=-12953.2788668 $\nu_{i=}$948

C	1.3464723	-0.9146894	-0.7233600
H	2.4355927	-0.7397127	-0.8248902
C	1.1649668	-2.1646211	0.2290973
H	1.0964148	-3.0438438	-0.4447283
C	0.7639306	-1.1742064	-2.1064382
C	-0.6322286	-1.2644379	-2.3253653
C	1.6116442	-1.3439106	-3.2161813
C	-1.1543406	-1.5107067	-3.5978954
H	-1.3347304	-1.1365426	-1.4855079
C	1.1055710	-1.5974127	-4.5032960
H	2.7018377	-1.2705707	-3.0781303
C	-0.2882430	-1.6817173	-4.7029287
H	-2.2387334	-1.5713565	-3.7709629
H	1.8059121	-1.7171082	-5.3406935
C	2.3456617	-2.4332565	1.1665232
C	3.0538595	-1.4102536	1.8278893
C	2.7134135	-3.7730830	1.4351066
C	4.0916907	-1.7058677	2.7289566
H	2.8329248	-0.3481365	1.6455836
C	3.7442953	-4.0812691	2.3258602
H	2.1805313	-4.5976972	0.9362013
C	4.4435217	-3.0464235	2.9884125
H	4.6152472	-0.8728371	3.2165025
H	4.0323126	-5.1229514	2.5294361
N	-0.0453491	-1.9983570	1.0489477

N	0.8043476	0.3389440	-0.1576676
O	-0.8891885	-1.9170997	-5.9019765
O	5.4306239	-3.4389785	3.8414884
C	6.1569092	-2.4397590	4.5410174
H	6.8911454	-2.9739862	5.1715673
H	6.7015198	-1.7611152	3.8476203
H	5.4957386	-1.8276127	5.1937508
C	-0.0737828	-2.0698426	-7.0531038
H	-0.7620243	-2.2405066	-7.9010425
H	0.5309499	-1.1581276	-7.2560965
H	0.6116178	-2.9422550	-6.9631118
C	1.6251698	1.4498244	0.0450292
O	2.8433618	1.3918531	0.0861613
C	0.8522569	2.7741318	0.1101184
C	0.0948505	3.1952926	1.2295252
C	0.9028709	3.5903792	-1.0432331
C	-0.6576165	4.3916217	1.1601353
C	0.2331580	4.8340701	-1.0848936
C	-0.5574087	5.2313059	0.0204365
S	0.0457357	2.1114496	2.7440957
O	-0.0187253	2.9775765	3.9306945
O	-1.1419858	1.1923024	2.4725955
O	1.2975629	1.2545436	2.6403533
C	-1.0581569	-2.8211166	1.0283915
C	-1.1976589	-3.9924942	0.1384759
C	-2.2128606	-2.4973090	1.9704571
C	-2.4498325	-4.7075167	0.0227738
H	-0.5939017	-3.9611437	-0.7844247
C	-3.1953922	-3.6544760	2.1873418
C	-3.6368667	-4.2603375	0.8525330
H	-4.1712481	-3.4903633	0.2477784
H	-4.3579184	-5.0919090	0.9893897
Li	0.1446462	-0.2538226	2.0437190
H	-0.1609010	0.5649432	-0.4205668
C	-2.1546272	-6.6149477	0.7768380
C	-0.8104874	-6.8748111	0.3752919
C	-3.2546340	-7.4727347	0.3012006
C	-0.5517427	-7.8796312	-0.6406132
C	0.7437322	-8.1565433	-1.1437186
C	-1.6578702	-8.6232400	-1.1325081
C	0.9285363	-9.1509032	-2.1050338
H	1.5888987	-7.5717343	-0.7531534
C	-1.4713746	-9.6284397	-2.0980378
C	-0.1817669	-9.8857164	-2.5780932
H	1.9352834	-9.3643510	-2.4926283
H	-2.3475793	-10.1868141	-2.4562899
H	-0.0359232	-10.6718444	-3.3342802
O	-2.9387099	-8.3965854	-0.7069616
O	-4.4084486	-7.4206372	0.6713805
O	0.1232801	-6.0830003	0.8094242
H	-0.3597486	-5.0195152	0.7303582

H	-2.6990907	-5.0652936	-0.9903003
H	-2.3019731	-6.2405421	1.7999568
Br	-1.8708112	4.8764489	2.5362093
Br	1.8526855	2.9787454	-2.5790576
Br	0.3726168	5.9441898	-2.6131857
Br	-1.4910422	6.8799657	-0.0417306
H	-1.8036042	-2.1301332	2.9331986
H	-2.7559458	-1.6302399	1.5301295
H	-2.7208088	-4.4404168	2.8174031
H	-4.0756297	-3.3004350	2.7599650

TS 14c pro (S)

E(SCF+ZPE)=-12953.2791288 $\nu_{i=}$787

C	-0.5889170	-2.8398049	-0.3537237
H	-0.0306061	-3.7975515	-0.2469838
C	-1.4778522	-2.6994291	0.9461743
H	-2.1916616	-3.5511212	0.8915265
C	-1.4759989	-2.9595176	-1.5845911
C	-1.8875688	-1.8688285	-2.3837667
C	-1.9998800	-4.2301796	-1.8877053
C	-2.7988978	-2.0483868	-3.4280146
H	-1.4526910	-0.8684562	-2.2413767
C	-2.9169717	-4.4283181	-2.9343312
H	-1.6781220	-5.1055459	-1.2992679
C	-3.3293533	-3.3262590	-3.7118634
H	-3.1048076	-1.2019310	-4.0593943
H	-3.2878907	-5.4414731	-3.1395590
C	-0.6512202	-2.8783433	2.2223820
C	-0.1373993	-1.7908254	2.9573112
C	-0.3402179	-4.1841378	2.6720511
C	0.6656237	-1.9874078	4.0963572
H	-0.3700714	-0.7591164	2.6509918
C	0.4520805	-4.3960964	3.8014373
H	-0.7349168	-5.0565653	2.1257596
C	0.9687057	-3.2959441	4.5261384
H	1.0444602	-1.1101517	4.6375772
H	0.6878115	-5.4108583	4.1536723
N	-2.1527360	-1.4063261	0.9059856
N	0.3670472	-1.7183009	-0.3363765
O	-4.2107075	-3.3961229	-4.7513887
O	1.7296727	-3.6013076	5.6118204
C	2.2872835	-2.5404453	6.3730388
H	2.8657982	-3.0138072	7.1872318
H	2.9704265	-1.9087568	5.7631809
H	1.5000149	-1.8925415	6.8186049
C	-4.7437445	-4.6606263	-5.1022784
H	-5.4178676	-4.4881226	-5.9615583
H	-3.9485580	-5.3778348	-5.4063578
H	-5.3302701	-5.1118839	-4.2699607
C	1.2132731	-1.3519450	-1.3897186
O	1.1076281	-1.7275669	-2.5401729

C	2.3765611	-0.4551646	-0.9202884
C	2.2533805	0.9198629	-0.6024908
C	3.6384326	-1.0821354	-0.8121676
C	3.3793642	1.6344836	-0.1283726
C	4.7867752	-0.3539656	-0.4303826
C	4.6534135	1.0114768	-0.0829806
S	0.5782379	1.7370493	-0.6991897
O	0.7560043	3.1285041	-1.1424205
O	-0.2290382	0.8612965	-1.6437659
O	0.0132850	1.5106690	0.7021207
C	-3.3615704	-1.2404398	1.3761512
C	-3.9040240	0.1248565	1.3839817
C	-4.2089811	-2.3587291	1.9596463
C	-5.1388928	0.4710785	2.0527353
H	-3.1341443	0.9173816	1.3342879
C	-5.6893532	-1.9906738	2.1400935
C	-5.8664082	-0.6266438	2.8122338
H	-5.4282410	-0.6492451	3.8366200
H	-6.9377919	-0.3712217	2.9398685
Li	-1.0478175	0.0096407	-0.0270787
H	0.7971866	-1.5914758	0.5907853
C	-6.4943679	0.9859464	0.6621514
C	-5.6940203	1.6487478	-0.3230989
C	-7.5996466	1.7147082	1.3198074
C	-5.8269854	3.0779535	-0.5126928
C	-4.9989860	3.8216306	-1.3916078
C	-6.8391261	3.7507989	0.2235398
C	-5.1858488	5.1964323	-1.5358341
H	-4.2129946	3.2864774	-1.9437693
C	-7.0323302	5.1343405	0.0717985
C	-6.2054800	5.8475307	-0.8054015
H	-4.5392274	5.7724736	-2.2132968
H	-7.8246046	5.6262538	0.6531871
H	-6.3532080	6.9320576	-0.9204619
O	-7.6538208	3.0980311	1.1113714
O	-8.4303234	1.2242772	2.0554519
O	-4.7392704	0.9727230	-0.8904266
H	-4.3002767	0.3546981	-0.0430425
H	-5.1353189	1.4513257	2.5586392
H	-6.7448691	-0.0647950	0.4529141
Br	3.7784640	-2.9573237	-1.1389886
Br	6.4794847	-1.2019425	-0.3613560
Br	6.1833286	1.9900062	0.4622714
Br	3.2230891	3.4088364	0.5293832
H	-4.1108817	-3.2706548	1.3353074
H	-3.7559241	-2.6226793	2.9426710
H	-6.1919356	-1.9836723	1.1467296
H	-6.2037550	-2.7775604	2.7276309

TS 14d pro (R)
E(SCF+ZPE)=-12953.2806147 $\nu_{i=}$920

C	0.9620879	-0.9363443	0.4732094
H	1.3334720	-0.6558932	1.4793434
C	-0.0903589	-2.0969278	0.7045342
H	0.5140365	-3.0292923	0.6451107
C	2.1384847	-1.3917736	-0.3774050
C	3.4503390	-1.3732703	0.1478264
C	1.9671342	-1.8371870	-1.7040314
C	4.5452437	-1.7855384	-0.6160359
H	3.6146149	-1.0248469	1.1797091
C	3.0574976	-2.2488804	-2.4893716
H	0.9569492	-1.8746889	-2.1419980
C	4.3600559	-2.2269404	-1.9456501
H	5.5662107	-1.7707129	-0.2074525
H	2.8798882	-2.5874755	-3.5190257
C	-0.7069841	-2.0800093	2.1046681
C	-1.7256768	-1.1827861	2.4802970
C	-0.2247870	-2.9818302	3.0817641
C	-2.2558928	-1.1883346	3.7805340
H	-2.1147819	-0.4385103	1.7697848
C	-0.7389834	-2.9954713	4.3823047
H	0.5795058	-3.6893720	2.8191290
C	-1.7657260	-2.0956776	4.7438193
H	-3.0416267	-0.4636130	4.0315218
H	-0.3627555	-3.6964154	5.1416826
N	-1.1066700	-2.0885807	-0.3579558
N	0.3826030	0.3003185	-0.1146561
O	5.4851038	-2.6064371	-2.6127377
O	-2.2115458	-2.1786047	6.0295603
C	-3.2261632	-1.2817697	6.4532382
H	-3.4249706	-1.5169643	7.5151543
H	-2.9020255	-0.2206278	6.3719216
H	-4.1661308	-1.4133124	5.8720066
C	5.3655889	-3.0480023	-3.9555106
H	6.3890398	-3.2926379	-4.2943502
H	4.7308799	-3.9584956	-4.0378648
H	4.9461783	-2.2571055	-4.6166965
C	0.1371072	1.4172090	0.6993389
O	0.0590006	1.3620595	1.9121425
C	0.0926003	2.7442687	-0.0740379
C	-1.0132131	3.1852010	-0.8416204
C	1.2469772	3.5557696	0.0118984
C	-0.9330009	4.4038307	-1.5560098
C	1.3024464	4.8155422	-0.6255751
C	0.2063011	5.2390344	-1.4149176
S	-2.4986006	2.0692609	-0.9859175
O	-3.7096951	2.8936507	-1.1080035
O	-2.1263279	1.1751912	-2.1652240
O	-2.4354449	1.1999281	0.2610460
C	-1.7326852	-3.1849603	-0.6939435
C	-1.5183150	-4.4995742	-0.0637901
C	-2.8016032	-3.0735360	-1.7716311

C	-2.3206342	-5.6505699	-0.4182714
C	-3.1146005	-4.4039679	-2.4678792
C	-3.4286564	-5.4975628	-1.4422474
Li	-1.6448940	-0.2323371	-0.8639714
H	0.7633775	0.5397625	-1.0384768
C	-1.0825678	-6.9882457	-1.3848471
C	0.1414028	-6.8208396	-0.6678566
C	-1.7528662	-8.3009132	-1.4160517
C	0.6258544	-7.8992231	0.1752024
C	1.7962408	-7.7939627	0.9674781
C	-0.1104407	-9.1138684	0.1857534
C	2.2201950	-8.8733440	1.7436560
H	2.3515991	-6.8452113	0.9462100
C	0.3184352	-10.2029921	0.9649670
C	1.4788662	-10.0764579	1.7380315
H	3.1284691	-8.7897924	2.3578602
H	-0.2727014	-11.1293634	0.9511420
H	1.8136373	-10.9287627	2.3484794
O	-1.2582613	-9.2794989	-0.5411532
O	-2.7148351	-8.5917027	-2.0951699
O	0.6977768	-5.6477611	-0.6613931
H	-0.2034265	-4.9181893	-0.5378958
H	-4.3445079	-5.2212398	-0.8690778
H	-3.6568249	-6.4689527	-1.9269024
H	-2.5530039	-6.3371012	0.4129659
H	-1.1862995	-6.4235843	-2.3222682
H	-1.1798426	-4.4620650	0.9860240
H	-3.7248380	-2.7024613	-1.2690735
H	-2.5149257	-2.2896438	-2.5020802
H	-2.2501008	-4.7128169	-3.0987597
H	-3.9660626	-4.2771711	-3.1657346
Br	2.7692054	2.9160702	0.9677316
Br	2.8344704	5.9137358	-0.4413622
Br	0.2817822	6.9162954	-2.2949017
Br	-2.2933094	4.9345120	-2.7680057

TS 14e pro (R)

E(SCF+ZPE)=-12953.2590114 $\nu_{i=}$836

C	-0.0923668	-1.3774842	-0.6266275
H	0.1212844	-0.3105860	-0.4282562
C	-1.4660145	-1.4028621	-1.4322776
H	-1.1607888	-1.1530280	-2.4742343
C	-0.1338847	-2.0778778	0.7257874
C	0.3348932	-3.3972422	0.9029350
C	-0.6649224	-1.4111191	1.8453833
C	0.2610574	-4.0309705	2.1456091
H	0.7829622	-3.9297268	0.0517311
C	-0.7510387	-2.0332237	3.1033166
H	-1.0228525	-0.3747704	1.7404187
C	-0.2863563	-3.3561741	3.2597331
H	0.6333166	-5.0561440	2.2874399

H	-1.1634591	-1.4718979	3.9527016
C	-2.0841203	-2.7935215	-1.4551372
C	-2.7871841	-3.3238836	-0.3541693
C	-1.9126554	-3.6234084	-2.5882296
C	-3.3149430	-4.6251825	-0.3776764
H	-2.9323948	-2.7085561	0.5458870
C	-2.4191321	-4.9265792	-2.6238635
H	-1.3845354	-3.2326070	-3.4738559
C	-3.1280273	-5.4417289	-1.5155728
H	-3.8579730	-4.9971223	0.5014860
H	-2.2854409	-5.5691301	-3.5060780
N	-2.2969624	-0.2821276	-0.9600439
N	0.9637367	-1.8437781	-1.5185794
O	-0.3119386	-4.0547005	4.4319451
O	-3.5908053	-6.7162176	-1.6393643
C	-4.2994178	-7.2922240	-0.5536288
H	-4.5694926	-8.3169712	-0.8678226
H	-3.6751296	-7.3481052	0.3656262
H	-5.2309588	-6.7293129	-0.3205807
C	-0.8143431	-3.4130640	5.5910842
H	-0.7346364	-4.1473441	6.4138771
H	-0.2211212	-2.5091643	5.8562854
H	-1.8814325	-3.1168668	5.4747580
C	2.0273489	-1.0916599	-1.9704731
O	2.6203127	-1.3343109	-3.0121853
C	2.6296396	-0.0588035	-0.9973969
C	2.4172353	1.3373442	-1.0643589
C	3.6242773	-0.5506136	-0.1145170
C	3.1988632	2.2255787	-0.2867775
C	4.3825793	0.3215179	0.6977766
C	4.1771382	1.7196962	0.6011563
S	1.0710475	2.0413819	-2.1145952
O	1.6920895	2.8876918	-3.1438765
O	0.2718343	0.8394478	-2.6162027
O	0.1480425	2.7332643	-1.1050647
C	-3.5139121	-0.0440366	-1.3844974
C	-4.1554617	1.1864931	-0.8845543
C	-4.2615585	-0.8709188	-2.4170072
C	-5.2949010	1.7889100	-1.5424805
H	-3.4717123	1.8721495	-0.3493802
C	-5.7034784	-0.4157246	-2.6884688
H	-3.6573088	-0.8125067	-3.3516670
H	-4.2321803	-1.9401787	-2.1323747
C	-5.8292241	1.1064892	-2.7895478
H	-6.0768324	-0.8949271	-3.6153934
H	-6.3670020	-0.7874095	-1.8753885
H	-6.8736907	1.4190129	-2.9900440
H	-5.2227501	1.4852754	-3.6451106
Li	-1.1566563	1.3643917	-1.2970318
H	0.7207070	-2.5938204	-2.1748389
H	-5.2865252	2.8911579	-1.5837422

C	-7.9105112	2.6286309	-0.7702899
C	-6.8841766	1.6746246	-0.3022603
C	-7.4655193	3.9883415	1.1987746
C	-7.7753430	5.1709271	1.8925325
C	-6.5400207	3.0551891	1.7389129
C	-7.1537279	5.4259787	3.1205735
H	-8.4959558	5.8722697	1.4489077
C	-5.9211011	3.3371836	2.9825751
C	-6.2242431	4.5132550	3.6682118
H	-7.3937425	6.3533907	3.6623577
H	-5.2034027	2.6057698	3.3813010
H	-5.7413972	4.7319045	4.6317161
H	-4.7713989	0.7924936	0.4078601
H	-7.0920635	0.6393903	-0.6096918
O	-8.0819349	3.7917586	-0.0073516
O	-5.3818916	0.9856330	1.3597144
C	-6.2824914	1.8452403	0.9839208
O	-8.5807357	2.5108129	-1.7736472
Br	3.9723173	-2.4227158	-0.0440139
Br	5.6780387	-0.3670564	1.8971503
Br	5.2136249	2.9014516	1.6617304
Br	2.9653088	4.1033002	-0.4245117

TS 14f pro (S)
E(SCF+ZPE)=-12953.2546479 $\nu_{i=}$1043

C	1.0824617	2.0084639	-0.7983011
H	0.1753909	2.5477104	-0.4752004
C	1.7218828	1.3331864	0.4499719
H	2.5814652	0.7131046	0.1233767
C	2.0017340	3.0394576	-1.4465384
C	3.3842097	2.8121369	-1.6366938
C	1.4514487	4.2369322	-1.9451349
C	4.1886358	3.7518722	-2.2879182
H	3.8542064	1.8878065	-1.2661325
C	2.2471132	5.1911243	-2.5997840
H	0.3693783	4.4092376	-1.8334666
C	3.6274396	4.9543614	-2.7739685
H	5.2656382	3.5821738	-2.4320217
H	1.7781553	6.1113629	-2.9734216
C	2.2404880	2.3499616	1.4711929
C	1.5205341	3.5171766	1.8021015
C	3.4288383	2.0817378	2.1882928
C	1.9624383	4.3928666	2.8113229
H	0.6038127	3.7916023	1.2515340
C	3.8849476	2.9455384	3.1866121
H	4.0090465	1.1740603	1.9572128
C	3.1547980	4.1120211	3.5114862
H	1.3758971	5.2965402	3.0250468
H	4.8140164	2.7409204	3.7386379
N	0.7224700	0.5215156	1.1782069
N	0.6510119	1.0459298	-1.8515301

O	4.4906736	5.8108783	-3.3904672
O	3.6772405	4.8908116	4.4977428
C	2.9958494	6.0830376	4.8567400
H	3.5923238	6.5513337	5.6608654
H	2.9218660	6.7909505	4.0016541
H	1.9719440	5.8758831	5.2403924
C	3.9823144	7.0293212	-3.9089789
H	4.8416099	7.5570137	-4.3623414
H	3.2098511	6.8574488	-4.6912568
H	3.5451143	7.6702005	-3.1107886
C	-0.6562845	0.7803104	-2.2807137
O	-0.9379228	0.7416089	-3.4695478
C	-1.6636092	0.1547975	-1.2958458
C	-2.5773170	0.8565389	-0.4764853
C	-1.7607531	-1.2613359	-1.3538490
C	-3.5151402	0.1571685	0.3211051
C	-2.7418144	-1.9630679	-0.6187145
C	-3.6242088	-1.2505767	0.2309809
S	-2.4849965	2.7142309	-0.4033697
O	-3.8554149	3.2525648	-0.4401947
O	-1.7790146	2.9178426	0.9527278
O	-1.5968184	3.0794080	-1.5479401
C	0.8022257	-0.7762055	1.2468884
C	1.7744399	-1.6003756	0.4818738
C	-0.1897856	-1.5042884	2.1370613
C	1.7793722	-3.0350789	0.6023281
H	1.9772891	-1.2180440	-0.5346388
C	0.3455323	-2.8406028	2.6717789
C	0.8146627	-3.7381916	1.5229373
H	-0.0577986	-4.0145196	0.8826316
H	1.2349844	-4.6987635	1.8870047
Li	-0.6331943	1.8543587	1.8628728
H	1.2104549	1.1817487	-2.7038916
C	3.6586590	-3.5193646	1.6090960
C	4.5007904	-2.5677783	0.9734742
C	3.8752063	-4.9607401	1.4474046
C	5.5061598	-3.0428156	0.0319952
C	6.3533912	-2.1715563	-0.6943870
C	5.6391866	-4.4449903	-0.1476590
C	7.3099617	-2.6856588	-1.5710813
H	6.2339859	-1.0890022	-0.5427370
C	6.6055534	-4.9645223	-1.0278853
C	7.4332672	-4.0835682	-1.7332876
H	7.9664725	-2.0064342	-2.1342741
H	6.6831271	-6.0545908	-1.1444527
H	8.1878382	-4.4903220	-2.4235302
O	4.8402488	-5.3455055	0.5029334
O	3.2697121	-5.8425437	2.0225114
O	4.2416323	-1.3048441	1.1185515
H	3.0269654	-1.2673932	0.9652814
H	2.0645177	-3.5961294	-0.3021679

H	3.1816352	-3.2345441	2.5561633
H	1.1846540	-2.6559868	3.3798660
H	-0.4411397	-3.3552859	3.2583271
H	-0.5044820	-0.8352417	2.9639844
H	-1.0988573	-1.7013919	1.5257380
Br	-0.5548771	-2.2489576	-2.4569590
Br	-2.8704135	-3.8541490	-0.7407324
Br	-4.9186702	-2.1880764	1.2529395
Br	-4.6086765	1.0767401	1.5766821

TS 14g pro (S)
E(SCF+ZPE)=-12953.2767354 $\nu_{i=}$865

C	1.3736375	-0.5070067	0.1695812
H	0.9420202	0.4237964	-0.2493460
C	2.1152099	-0.1198642	1.5009553
H	2.7665326	-0.9858950	1.7671118
C	2.3566936	-1.0812484	-0.8425167
C	2.9627052	-2.3436847	-0.6483334
C	2.7042218	-0.3552984	-1.9940062
C	3.8784560	-2.8578171	-1.5681812
H	2.7023609	-2.9540659	0.2320692
C	3.6205461	-0.8562085	-2.9343288
H	2.2476384	0.6315949	-2.1692064
C	4.2152992	-2.1175137	-2.7253096
H	4.3455876	-3.8429665	-1.4241093
H	3.8559313	-0.2573600	-3.8241428
C	3.0236021	1.0874620	1.2748240
C	2.4717531	2.3569569	1.0049040
C	4.4297180	0.9666644	1.3199510
C	3.2932904	3.4766426	0.7946211
H	1.3781557	2.4822398	0.9422253
C	5.2602631	2.0718861	1.1093015
H	4.8861776	-0.0178614	1.5112181
C	4.6982284	3.3414374	0.8475894
H	2.8246716	4.4473092	0.5835479
H	6.3556980	1.9789698	1.1375419
N	1.1367619	0.1275200	2.5709886
N	0.2349074	-1.3896826	0.4921663
O	5.1095550	-2.7030110	-3.5698352
O	5.5828923	4.3606941	0.6587244
C	5.0768647	5.6568026	0.3809361
H	5.9569696	6.3154512	0.2635309
H	4.4819571	5.6776241	-0.5591880
H	4.4456370	6.0431708	1.2121945
C	5.4625306	-2.0208941	-4.7625417
H	6.1796373	-2.6736075	-5.2934580
H	4.5791962	-1.8466720	-5.4162827
H	5.9519839	-1.0433594	-4.5526432
C	-0.8514160	-1.7263654	-0.3227296
O	-1.5598435	-2.6870219	-0.0753858
C	-1.0570895	-0.8721302	-1.5817159

C	-1.6127281	0.4307265	-1.5785671
C	-0.6963490	-1.4660848	-2.8149318
C	-1.6817479	1.1743111	-2.7807650
C	-0.8565445	-0.7667645	-4.0329888
C	-1.3313730	0.5665910	-4.0128913
S	-2.1920327	1.1706122	0.0262747
O	-2.4156787	-0.0187583	0.9468327
O	-0.9524135	1.8995276	0.5496758
O	-3.3877205	1.9815867	-0.2489889
C	1.4839103	0.0799262	3.8307296
C	0.4578035	0.4104224	4.8350550
C	2.8842921	-0.2479635	4.3152285
C	0.7739979	0.6015677	6.2325084
H	-0.3858719	1.0059253	4.4406639
C	2.9814856	-0.5467671	5.8182893
C	2.2268763	0.4872748	6.6587699
H	2.6804515	1.4958022	6.5160933
H	2.3015879	0.2663869	7.7429503
Li	-0.7021209	0.3602901	1.8096946
H	0.4031813	-2.1296437	1.1843174
C	-0.0729347	-0.9215256	7.2890663
C	-1.2870429	-1.1510076	6.5692996
C	-0.1190624	-0.5382211	8.7141305
C	-2.5570249	-0.8214329	7.1853571
C	-3.7980621	-0.9426090	6.5105507
C	-2.5404640	-0.3667730	8.5316263
C	-4.9875598	-0.6253282	7.1671018
H	-3.7907847	-1.2898749	5.4672029
C	-3.7394080	-0.0531162	9.1945439
C	-4.9536951	-0.1841435	8.5092674
H	-5.9490853	-0.7174445	6.6416728
H	-3.6941717	0.2942165	10.2362062
H	-5.8924974	0.0638123	9.0272421
O	-1.3756324	-0.2036641	9.2345136
O	0.8382438	-0.4482564	9.4540729
O	-1.2018178	-1.4843179	5.3159131
H	-0.3534633	-0.8159467	4.9172433
H	0.2043062	1.3970530	6.7411843
H	0.7680187	-1.5888627	7.0514537
H	3.3038628	-1.0870067	3.7210711
H	3.5192547	0.6286731	4.0521878
H	4.0458703	-0.5868014	6.1245579
H	2.5736772	-1.5621431	6.0240347
Br	-2.1593191	3.0117913	-2.7802535
Br	-1.4901876	1.5315727	-5.6380821
Br	-0.4176220	-1.5984835	-5.6782722
Br	0.0229259	-3.2275341	-2.8360639

TS 14h pro (R)
E(SCF+ZPE)=-12953.24875 $\nu_{i=}$826

C	0.3303679	2.4525980	-0.8476142

H	-0.7128433	2.7450842	-0.6333901
C	0.9975487	2.0026784	0.4877604
H	2.0160765	1.6315658	0.2510672
C	1.0099872	3.6694594	-1.4679381
C	2.4160900	3.7828266	-1.5655032
C	0.2221689	4.6850962	-2.0437381
C	3.0122446	4.8760360	-2.2008624
H	3.0696142	3.0055676	-1.1390350
C	0.8060774	5.7904232	-2.6836865
H	-0.8739118	4.5867882	-2.0117343
C	2.2109791	5.8939749	-2.7651602
H	4.1054763	4.9695504	-2.2755459
H	0.1540789	6.5584253	-3.1213276
C	1.1155923	3.1508877	1.4901112
C	0.0207271	3.9921683	1.7821028
C	2.3143039	3.3645987	2.2062129
C	0.1063108	5.0003408	2.7581364
H	-0.9279300	3.9028557	1.2233891
C	2.4194375	4.3653091	3.1759121
H	3.1963775	2.7410398	1.9893311
C	1.3111241	5.1925131	3.4669459
H	-0.7689710	5.6373589	2.9431435
H	3.3572884	4.5351551	3.7246108
N	0.1766532	0.9440839	1.1129905
N	0.2781050	1.3848149	-1.8776646
O	2.8808676	6.9201202	-3.3635410
O	1.5042311	6.1376728	4.4282153
C	0.4277451	7.0017110	4.7584840
H	0.8019807	7.6750335	5.5512941
H	0.1054032	7.6143356	3.8872636
H	-0.4508568	6.4398749	5.1471775
C	2.1276026	7.9619083	-3.9622264
H	2.8611504	8.6730177	-4.3849290
H	1.4760906	7.5852144	-4.7820676
H	1.4936670	8.4962660	-3.2195768
C	-0.8273171	0.6993096	-2.3654492
O	-0.9463497	0.4328906	-3.5546261
C	-1.7563033	-0.0628487	-1.3979324
C	-2.9793826	0.4162814	-0.8700923
C	-1.4302990	-1.4310963	-1.2069947
C	-3.8308501	-0.4446444	-0.1337836
C	-2.3011877	-2.3116692	-0.5308377
C	-3.5160061	-1.8184368	0.0054364
S	-3.3416742	2.2453055	-0.9843011
O	-4.7784739	2.4523197	-1.2301494
O	-2.8850087	2.6651732	0.4325921
O	-2.4072080	2.7430904	-2.0361916
C	0.7199629	-0.1462339	1.5933162
C	2.1537746	-0.4491287	1.6425289
C	-0.1950273	-1.1909197	2.2048561
C	2.6526118	-1.6287650	2.3299329

C	0.3918403	-1.7275671	3.5185194
C	1.7307355	-2.4345068	3.2590777
Li	-1.6961540	1.6342146	1.3566859
H	0.8779218	1.6103413	-2.6811423
C	3.1625810	-2.9513051	0.9309643
C	3.9369245	-2.1290280	0.0490162
C	3.7938781	-4.1237405	1.5710533
C	5.3741376	-2.2936201	-0.0089054
C	6.2184385	-1.4627692	-0.7884596
C	5.9499946	-3.3451917	0.7544803
C	7.5963236	-1.6782849	-0.8054974
H	5.7545921	-0.6550753	-1.3728550
C	7.3388263	-3.5636687	0.7344726
C	8.1521143	-2.7303572	-0.0426357
H	8.2491595	-1.0332841	-1.4113427
H	7.7536411	-4.3859146	1.3342260
H	9.2391287	-2.9015470	-0.0567526
O	5.1969741	-4.1782337	1.5353502
O	3.2127300	-5.0097360	2.1581027
O	3.3413433	-1.1346484	-0.5392368
H	2.5894624	-0.7645468	0.2271442
H	0.5322071	-0.8835938	4.2281665
H	-0.3155263	-2.4346153	3.9969921
H	2.2689760	-2.6097657	4.2125730
H	1.5436264	-3.4500773	2.8528858
H	-1.2073842	-0.7626920	2.3522387
H	-0.3191608	-2.0310438	1.4845257
H	3.6702261	-1.4945990	2.7333299
H	2.1229112	-3.1435847	0.6239411
H	2.8345382	0.4165330	1.6409768
Br	-5.3902337	0.2186847	0.7309862
Br	-4.6904997	-2.9931919	0.9213210
Br	-1.8402741	-4.1402777	-0.3087924
Br	0.2364130	-2.0914241	-1.8592261

Single-point energies with solvent correction employing COSMO as implemented in TURBOMOLE 6.3. Settings were applied as default with epsilon=7.25 for THF. Radii were optimized.

Stationary point	Total energy COSMO-TPSS/SVP//TPSS/SVP [kcal/mol]	Relative energy [kcal/mol]
**TS 14 a**	-12952.64965	0.3
**TS 14 b**	-12952.64769	1.5
**TS 14 c**	-12952.64214	5.0
**TS 14 d**	-12952.65015	0.0

**TS 14 e**	-12952.63241	11.1
**TS 14 f**	-12952.62943	13.0
**TS 14 g**	-12952.64859	1.0
**TS 14 h**	-12952.62333	16.8
**Stationary point**	**Total energy COSMO-BP86/SVP//BP86/SVP [kcal/mol]**	**Relative energy [kcal/mol]**
**12**	-1969.34240	0.0
**13**	-1969.27485	42.4
**14**	-1969.28531	35.8
**15**	-1969.32593	10.3
**16**	-1969.33962	1.7
